# Bioprinted Hydrogels as Vehicles for the Application of Extracellular Vesicles in Regenerative Medicine

**DOI:** 10.3390/gels11030191

**Published:** 2025-03-08

**Authors:** Marta Camacho-Cardenosa, Victoria Pulido-Escribano, Guadalupe Estrella-Guisado, Gabriel Dorado, Aura D. Herrera-Martínez, María Ángeles Gálvez-Moreno, Antonio Casado-Díaz

**Affiliations:** 1 Unidad de Gestión Clínica de Endocrinología y Nutrición-GC17, Instituto Maimónides de Investigación Biomédica de Córdoba (IMIBIC), Hospital Universitario Reina Sofía, 14004 Córdoba, Spain; marta.camacho@imibic.org (M.C.-C.); victoriapulido7@gmail.com (V.P.-E.); z42esgug@uco.es (G.E.-G.); aurita.dhm@gmail.com (A.D.H.-M.); 2 Departamento Bioquímica y Biología Molecular, Campus Rabanales C6-1-E17, Campus de Excelencia Internacional Agroalimentario (ceiA3), Universidad de Córdoba, 14071 Córdoba, Spain; bb1dopeg@uco.es; 3 CIBER de Fragilidad y Envejecimiento Saludable (CIBERFES), 14004 Córdoba, Spain

**Keywords:** 3D bioprinting, extracellular vesicles, tissue engineering, regenerative medicine, hydrogels, bioinks

## Abstract

Three-dimensional bioprinting is a new advance in tissue engineering and regenerative medicine. Bioprinting allows manufacturing three-dimensional (3D) structures that mimic tissues or organs. The bioinks used are mainly made of natural or synthetic polymers that must be biocompatible, printable, and biodegradable. These bioinks may incorporate progenitor cells, favoring graft implantation and regeneration of injured tissues. However, the natures of biomaterials, bioprinting processes, a lack of vascularization, and immune responses are factors that limit the viability and functionality of implanted cells and the regeneration of damaged tissues. These limitations can be addressed by incorporating extracellular vesicles (EV) into bioinks. Indeed, EV from progenitor cells may have regenerative capacities, being similar to those of their source cells. Therefore, their combinations with biomaterials can be used in cell-free therapies. Likewise, they can complement the manufacture of bioinks by increasing the viability, differentiation, and regenerative ability of incorporated cells. Thus, the main objective of this review is to show how the use of 3D bioprinting technology can be used for the application of EV in regenerative medicine by incorporating these nanovesicles into hydrogels used as bioinks. To this end, the latest advances derived from in vitro and in vivo studies have been described. Together, these studies show the high therapeutic potential of this strategy in regenerative medicine.

## 1. Introduction

In the history of humanity, one of the main objectives of medicine has been to replace diseased or damaged parts of the human body. Organs are biological structures that are easily damaged and deteriorate with aging, which directly affects the health of the organism. In the last century, there have been very important medical advances in the field of organ transplantation. However, there are still major difficulties in their development and general use due to ethical aspects, organ shortage, and graft rejection problems [1,2]. In an attempt to overcome these limitations, tissue engineering has been proposed as a possible solution.

One of the main aspects in tissue engineering is the use of different materials to manufacture structures that promote tissue regeneration. For thousands of years, man has used different types of materials for therapeutic purposes. For example, the Egyptians, 4000 years ago, used linen threads for wound healing. For the same purpose, Europeans used catgut in the Middle Ages [3]. On the other hand, the Incas used gold plates for the treatment of cranial fractures, whereas the Mayans made artificial teeth from seashells, capable of osseointegration [4,5]. More recently, between the 16th and 19th centuries, metals were used as substitutes for hard tissues. In the middle of the last century, bioinert materials such as titanium and the first polymers began to be used. In the 1970s, bioactive and biodegradable materials based on ceramic compounds and polymers appeared. Already in this century, in the last decade, biomaterials have been developed that, together with cells, can be bioprinted into biocompatible three-dimensional biological structures that mimic organs and tissues [3].

Currently, polymer-based materials are among the most widely used for the manufacture of biomaterials. The first plastic (celluloid) was manufactured in 1860, from which other similar materials were later derived [6]. But it was not until 1939 that the first polymer was used as a material to be implanted. That material was cellophane, being used to envelop blood vessels in the treatment of aneurysms [7]. Since then, the number of new polymers with diverse physicochemical and bioactive properties used in the manufacture of biomaterials for tissue regeneration has grown enormously.

The concept of biomaterials began in the last century. One of the first definitions of biomaterial was that of orthopedic surgeon Jonathan Cohen in 1967. He defined them as “all materials that are used as implants, with the exception of drugs and soft biological tissues” [8]. Subsequently, in 1982, during the “National Institutes of Health Consensus Development Conference Statement on the Clinical Applications of Biomaterials” (Bethesda, MD, USA), biomaterial was defined as “A substance (other than a drug) or combination of substances, of synthetic or natural origin, that can be used for any period of time, as a whole or as part of a system that treats, augments or replaces any tissue, organ or function of the body” [3]. In the 1980s, biomaterials began to be used as a support for the culture and growth of cells, to promote tissue regeneration. Following these advances, the term “tissue engineering” appeared at that time. This was defined by the National Science Foundation in 1988 as “the application of engineering and life science principles and methods to the fundamental understanding of structure-function relationships in normal and pathological mammalian tissues and the development of biological substitutes to restore, maintain, or improve tissue function” [9].

Furthermore, tissue engineering in the late 1990s gained prominence with the emergence and development of regenerative medicine. Regenerative medicine was defined as “an emerging interdisciplinary field of research and clinical applications focused on the repair, replacement or regeneration of cells, tissues or organs to restore impaired function resulting from any cause, including congenital defects, disease, trauma and aging. It uses a combination of several technological approaches that moves it beyond traditional transplantation and replacement therapies. These approaches may include, but are not limited to, the use of soluble molecules, gene therapy, stem cell transplantation, tissue engineering and the reprogramming of cell and tissue types” [10].

Since then, stem cell research, as well as the emergence of new biomaterials and new technologies in the field of tissue engineering, has continued to grow. This has now led to the development of 3D bioprinting as the most promising technology for the manufacture of artificial tissues and organs. This technology allows the creation of biological structures using bioinks composed of biomaterials and living cells, distributed according to a previous design, which can be implanted to regenerate damaged tissue. Bioprinting can be used to create complex scaffolds with specifically designed pores and channels. This way, they allow cell adhesion, proliferation, and growth. In addition, these biomaterials are generally designed to degrade after implantation, being replaced by new regenerated tissue. The implantation of artificial organs generated by 3D bioprinting would significantly reduce graft rejection and the use of immunosuppressive therapies that otherwise may produce significant side effects in patients [11]. Thus, the goal of 3D bioprinting is the large-scale production of functional human tissues and organs. However, at present, there are still important limitations in bioprinting related to the biological and biomechanical characteristics of the biomaterials used as well as the need to be able to use multiple cell types in the bioink in a viable manner, maintaining a microenvironment that allows them to proliferate and exert their vital functions.

Cell therapy, together with tissue engineering and the development of bioprinting, represent breakthroughs in regenerative medicine. Thus, mesenchymal stem cells (MSC) with the ability to differentiate into different cell types and induce regenerative processes are one of the main cell types used in bioinks for 3D bioprinting, aimed at different applications [12,13]. However, following the discovery that transplanted MSC exert their effects mainly through the secretion of paracrine factors, the last decade has seen major advances in the development of cell-free therapies applied to regenerative medicine [14]. Indeed, numerous studies have shown that the secretome produced by MSC possesses proangiogenic, immunomodulatory, anti-inflammatory, antiapoptotic, antifibrotic, and proliferative properties [15].

Forming part of the secretome are extracellular vesicles (EV). They can transport nucleic acids, proteins, lipids, and other metabolites involved in cellular communications. EV are involved in numerous physiological and pathological processes such as the maintenance of homeostasis, regulation of the immune system, tissue regeneration, and tumor formation [14].

It should be noted that the use of cell therapies may have several drawbacks. Among them are (i) decreased cell viability and regenerative capacity of implanted cells; (ii) possible immune histocompatibility problems; and (iii) the possibility to generate tumors. All that limits their use in regenerative medicine. In this context, the use of EV may be an alternative because they can potentially replicate the regenerative effects of the stem cell without their potential negative side effects [16]. Indeed, numerous preclinical trials have demonstrated the therapeutic potential of MSC-derived EV for treatment of different pathologies, like cardiac lesions, skin lesions, and liver, neuronal, pulmonary, and renal diseases, among others [17].

New advances in the manufacture of bioinks for 3D bioprinting include the incorporation of EV with regenerative capacity. In numerous preclinical applications, the positive effect of EV-loaded scaffolds on different tissue regeneration (bone, skin, vascular, liver, etc.) has been observed [18]. Thus, the first part of this review shows the main tools and bioink characteristics that are currently being used in 3D bioprinting. The second part highlights the advances and studies carried out so far in the incorporation of biomaterials loaded with EV that allows the creation of structures by bioprinting, which have been functionally evaluated by in vitro and/or in vivo assays.

## 2. Bioprinting in Regenerative Medicine

The use of tissue engineering in regenerative medicine aims to form biological structures with cells and biomaterials that replace or help regenerate damaged tissues [19]. Initially, bioprinting was used for the creation of structures without biocompatibility requirements, for educational or surgical planning purposes. It has also been used for the fabrication of prostheses. These are biocompatible but not biodegradable elements. Another advance has been the construction of biocompatible and biodegradable structures. In the latter scaffolds produced by 3D printing, cells have been implanted once the supporting structures have been manufactured. However, this required sterilization of such structures, which could alter the chemical and mechanical properties of the scaffolds [20]. In addition, cell implantation processes may not allow homogeneous cell populations, or at least with characteristics similar to those of the damaged tissue [21], to be obtained.

**Figure 1:**
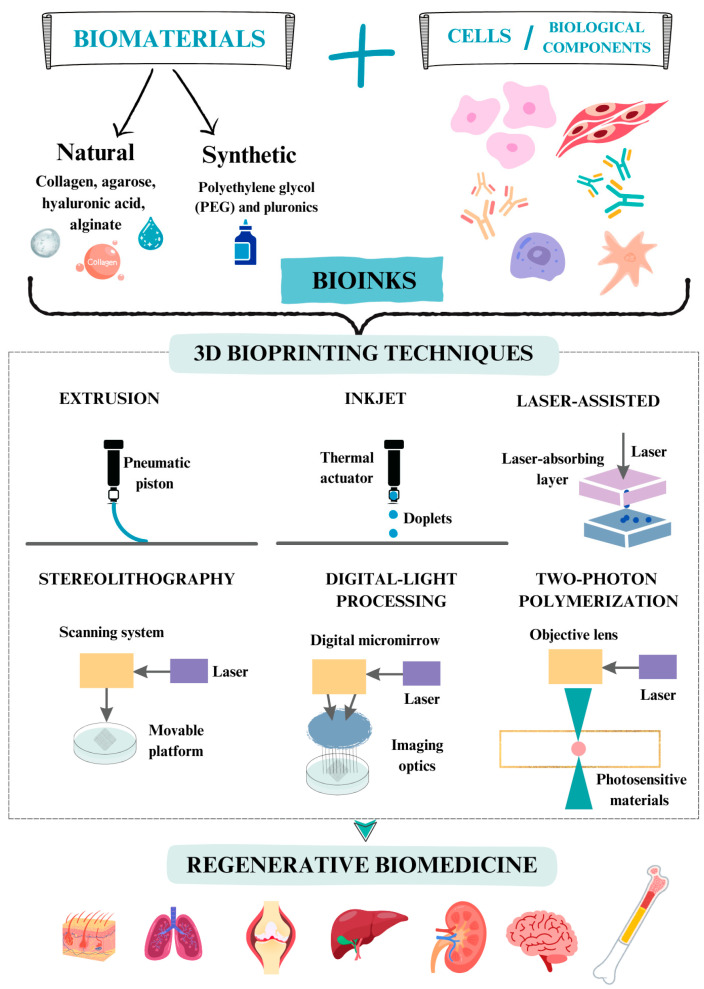
Bioprinting technology in regenerative medicine. Natural and synthetic biomaterials can be integrated with biological components to formulate bioinks. These bioinks, using different 3D bioprinting techniques, will be used for scaffolds with applications in regenerative medicine.

The solution to these limitations in the fabrication of three-dimensional (3D) biological structures has been accomplished in part with the development of 3D bioprinting. Such technology emerges as an intersection between different technologies and disciplines, including biology, chemistry, mechanical engineering, and informatics. Indeed, 3D bioprinting represents a new strategy to regenerate damaged human tissues by manufacturing complex structures from living cells using biocompatible and biodegradable biomaterials. With 3D bioprinting, it is possible to spatially deposit components of living tissues, such as extracellular matrix (ECM), growth factors, cells, etc., that form tissue-like structures (Figure 1). Four phases can be distinguished in 3D bioprinting: (i) acquisition of the necessary data to create the 3D model, supported by the use of computer-aided design (CAD) software; (ii) selection of the biomaterial, cell type, and other suitable biological compounds, which will constitute the bioink, according to the structure to be manufactured and the bioprinting technique to be used; (iii) bioprinting; and (iv) testing the functionality of the created structure [22].

## 3. 3D Bioprinting Technologies

Depending on the technique used, 3D bioprinting can be divided into four types: extrusion, inkjet, laser-assisted, and vat photopolymerization (Figure 1) [22].

### 3.1. Extrusion-Based Bioprinting

Extrusion-based bioprinting involves the application of bioinks in the form of continuous microfilaments. That is accomplished through a nozzle by either mechanical or pneumatic actuations. Depending on the operating mode, extrusion bioprinting can be classified as pneumatic when it is based on the use of compressed air or mechanically driven piston and screw. In the latter, the principle is similar to the piston-driven one but a screw connected to the motor performs the extrusion instead of the piston. Mechanical devices are more suitable for the extrusion of high-viscosity biomaterials. However, screw-driven devices can have negative effects on the cells loaded in the bioink due to higher pressure. Extrusion-based bioprinting is the most widely used method due to its versatility and affordability [23]. Its main advantage is its ability to print different types of biocompatible materials, with or without embedded cells.

Extrusion bioprinting allows the use of biomaterials with high viscosity, forming structures with high strength and mechanical capabilities as well as biomaterials of lower viscosity, providing a suitable environment for maintenance of cell viability and functionality. Thus, materials with viscosities between 30 and 6 × 10^7^ mPa/s can be used with this technology [24]. These biomaterials also normally have a thermally crosslinked mechanism. This allows them to be fluid at room temperature and therefore easily extruded, while they gel at temperatures close to physiological human body temperatures (35 to 40 °C). A limitation of extrusion bioprinting is that its resolution is more limited than in other technologies, being about 100 μm [25]. Additionally, due to the shear force produced during extrusion, cell viability may be compromised. Thus, depending on the pressure exerted and the nozzle diameter, cell viability ranges from 40 to 86% [26].

### 3.2. Inkjet 3D Bioprinting

Inkjet 3D bioprinting was the first technology used for bioprinting [27]. Its mechanism is based on the ejection of bioink droplets on demand, which will form the 3D structure. For this, the droplets must gel through crosslinking mechanisms by chemicals, pH, or ultraviolet light, which can slow down the process and decrease cell viability. The droplets in inkjet 3D bioprinting are directed to a specific position on the substrate. Then, the interaction between the bioink droplet and the substrate takes place. This process can be regulated by different forces such as piezoelectric, thermal, or electrostatic [22]. The fact that direct contact with the deposition surface is not required in the bioprinting process allows the use of heterogeneous mixtures of biomaterials and cells. This also increases the viability of encapsulated cells and increases both the speed and resolution of such printing. However, its main limitation is the need to use low-viscosity bioinks. This results in structures with low mechanical strength and low cell density [28]. Thus, their applications in regenerative medicine are restricted [27].

### 3.3. Laser-Assisted Bioprinting

Laser-assisted bioprinting (LAB), like inkjet bioprinting, does not require direct contact with the deposition surface. The basic elements of this type of bioprinter are (i) a pulsed laser beam; (ii) a focusing system; (iii) a “ribbon” as a donor layer that has an energy-absorbing layer with the ability to respond to laser stimulation; (iv) a container of liquid bioink solution; and (v) a substrate where the bioink will be directed and crosslinking will occur. In this technology, a laser is directed at the bioink droplet, heating it and moving it toward the deposition surface. This manufacturing method does not cause damage to the cells due to the lack of contact with the substrate. This technology enables the bioprinting of biomaterials with a high range of viscosity and cell density [29]. However, bioprinters using this technology have a high cost. In addition, if the device contains a metallic laser absorption layer, evaporation of this material and the appearance of metallic residues in the fabricated structures can occur with possible cytotoxic effects [24]. Although LAB allows high cell densities to be incorporated into the bioink, the heat produced in the droplet during the process may adversely affect cell viability [29,30].

### 3.4. Vat Photopolymerization (VP)-Based Bioprinting

Three methodologies are distinguished in this type of 3D bioprinting: stereolithography (SLA), digital light processing (DLP), and two-photon polymerization (TPP) [31], as described below.

#### 3.4.1. Stereolithography

Bioprinting by stereolithography (also known as the light-curing process) is based on a traditional printing method. In fact, it was the first commercial 3D printing technology, invented by Charles W. Hull in 1984. However, SLA technology is currently being applied more to scaffold printing than to the bioprinting of cell-loaded bioink. The process in SLA consists of a light-curing liquid solution being poured onto a mold of the desired structure. When the liquid solution acquires the predesigned shape, it is irradiated with laser or ultraviolet light to induce gelation of the bioink. The polymerization reaction requires the incidence of a single photon on the photosensitive resin to solidify the bioink. This process is fast, with irradiation with ultraviolet light of appropriate wavelength (325 to 355 nm) and intensity (10 to 400 mW) [32]. Bioprinting by SLA is characterized by a high resolution but it is quite slow. This is due to the fact that the process has to alternate cycles of bioink injection with irradiation-curing phases. In addition, the use of laser and UV irradiation can negatively affect the viability and functionality of cells incorporated in the bioink [33,34].

#### 3.4.2. Digital Light Processing

The DLP process is in part similar to SLA but the former uses micromirrors, allowing the gelation of an entire layer instead of dot by dot. These bioprinters use a container with photocurable bioink or photosensitive resin, gelling when irradiated with light of a specific wavelength (usually UV light). Through a platform, the gelled bioink is displaced vertically, allowing exposure of another new layer to the light beam. This process is repeated until the desired structure is formed. Due to the layer-by-layer manufacturing offered by DLP, the speed of bioprinting is superior to other technologies such as extrusion, inkjet, or SLA. However, DLP has a higher cost than SLA. In addition, the size of the bioprinted structures is generally limited. Moreover, the photosensitive resins used by DLP are generally toxic. This, together with the reduced range of bioinks available for this technology, limits the applications of 3D bioprinting with this technology in regenerative medicine [35].

#### 3.4.3. Two-Photon Polymerization

TPP, unlike SLA and DLP, which are based on single-photon absorption, is based on the induction of photopolymerization of bioinks by the absorption of two photons at the same time. This process occurs mainly in the super-strong laser focal point generated by a pulsed femtosecond laser. TPP technology allows a higher resolution than SLA and DLP, being able to reach nanometer and submicroscale scales. This is due to the ability of the device to move the absorption focus to the nanoscale in the photosensitive biomaterial. The disadvantage of TPP bioprinting is the scarcity of bioinks compatible with this technology. In addition, the use of laser and UV light can have toxic effects on the cells incorporated into the bioinks, which limits its applications [36].

## 4. Bioinks in 3D Bioprinting

### 4.1. Main Properties of Bioinks for Their Use in Regenerative Medicine

The result of 3D bioprinting must be a structure based on biocompatible biomaterials with a controllable degradation rate. In addition, it must exhibit mechanical properties similar to the tissue where it will be applied. Additionally, it should have low immunogenicity and the capacity to create suitable microenvironments for cell viability and functionality. To this end, the physicochemical and biological properties of bioinks are fundamental. In optimizing their manufacture, it is essential to take into account that they must retain cell viability and functionality before, during, and after 3D bioprinting. Bioinks can be defined as a mixture of biomaterials and living cells that mimic the characteristics of the extracellular matrix and allow cell proliferation, adhesion and differentiation [34]. In this sense, the functionality and applicability of bioinks is mainly influenced by three factors: biocompatibility, printability, and mechanical properties.

Biocompatibility is the property to simulate the environment of cells to facilitate their viability, functionality, and interconnection so that among the components of the bioink, there cannot be potentially cytotoxic compounds. Printability is the ability to form the desired structures, depending on the bioink’s viscosity, speed, and mode of gelation. Other parameters related to printing should also be considered, such as temperature, which must be within physiological ranges. Additionally, mechanical properties define the strength of the biomaterial once bioprinted and therefore its possible implantation in the tissue to be regenerated [22]. On the other hand, the structures formed by bioprinting must allow cell adhesion, proliferation, and differentiation. For this, it is essential that the fabricated structure possesses adequate morphology, porosity, and connectivity [37]. In addition to these characteristics, other important properties that bioinks intended for 3D bioprinting should have been appropriate viscoelastic parameters, fast gelling, nonimmunogenic, low cost, and the ability to transport or diffuse nutrients, oxygen, and waste [34,38].

The viscoelasticity and consistency of bioinks will depend on their rheological properties. These include viscosity, which indicates the resistance of a bioink to flow when a stress is applied, and the elastic limit defined as the maximum stress that the material can support without deformation. These properties should allow the maintenance of the structure adopted by the hydrogel once bioprinted. In addition, they will influence cell encapsulation, bioprintability, biocompatibility, and the resolution of bioprinting [39]. On the other hand, the gelatinization process of bioinks determines their solidification after extrusion or deposition on the structures. This crosslinking process must be fast, nontoxic to cells and not adversely affect printed structures. For this objective, depending on the purpose and type of bioprinting, biomaterials that gel in response to different physicochemical stimuli can be used. They include, for example, photosensitive, thermosensitive, enzyme-sensitive, pH-sensitive, or ion-sensitive biomaterials [22].

An important property of bioinks is that they should not elicit immune responses after implantation in vivo. Yet, in principle, biomaterials and biological components of bioinks are potential antigenic sources. This may induce the innate immune system to isolate the implanted materials by means of fibrotic capsules or the acquired immune system by provoking antigen-specific reactions. Therefore, this should be avoided in order not to induce a rapid degradation of scaffolds or processes of fibrosis instead of tissue regeneration [40]. Biodegradation is another important property of bioinks. The resulting scaffolds should be biodegradable and replaced by the extracellular matrix once inserted into the host tissue. Additionally, the resulting scaffold-degradation products should be nontoxic and should be easily metabolized and excreted, which will partly define the biocompatibility of bioinks [38]. This is directly related to the biosafety of hydrogels. Therefore, studies that assess the possible toxicity and immunogenicity of the metabolites generated during biodegradation should be considered [41]. Thus, for example, nanocellulose has been developed for bioprinting with increased biosafety by reducing the levels of immunogenic contaminants [42].

As has been described in this section, there are numerous parameters that affect the composition of bioinks to obtain the expected results. Optimization of these parameters is often carried out empirically, which is sometimes a slow and laborious process. However, currently, the development of artificial intelligence (AI), including machine learning (ML), allows the development of computational algorithms for such purposes. This can significantly accelerate the optimization of bioink manufacturing for 3D bioprinting [43]. This means that the new generation of bioinks must be characterized by high printability and biocompatibility [44]. This may lead to a considerable improvement in the application of this technology in regenerative medicine in the coming years.

### 4.2. Composition and Type of Bioinks

Bioinks are composed of biomaterials, cells, and/or factors with biological activity.

The National Institutes of Health (NIH) defines biomaterials as “any substance or combination of substances, other than drugs, of synthetic or natural origin, that can be used for any period of time, that augments or replaces part or all of any tissue, organ or function of the body, in order to maintain or improve the quality of life of the individual” [45]. Biomaterials used for the fabrication of bioinks include hydrogels, microcarriers, decellularized matrix components, cell pellet spheroids, nanocomposites, etc. [46]. Hydrogels have physical characteristics similar to those of the extracellular matrix, exhibiting a good ability to absorb water. Thus, they are the most common materials used for the manufacture of bioinks [47]. Depending on their origin, hydrogels can be natural (biosynthetic) or artificial (synthetic). The former are generated from biological tissues, such as collagen, agarose, hyaluronic acid (HA), alginate, etc. The latter are synthesized by artificial chemical methods. Among the most common are polyethylene glycol (PEG) and Pluronics F127.

The intrinsic properties of natural hydrogels favor cell proliferation, differentiation, and migration. However, as a basis for the creation of bioinks, they have the disadvantage of lower mechanical resistance compared to synthetic hydrogels. Among the biomaterials of natural origin, alginate is one of the most widely used in bioinks due to its easy gelation by ionic crosslinking, good printability, and mechanical properties. In fact, it has been commonly used together with other natural compounds, such as gelatin, collagen, and gelatin methacryloyl (GelMA), among others, for the manufacture of bioinks [48,49]. Collagen is one of the most important proteins in mammalian extracellular matrix formation. Although it is used in the composition of bioinks, it is usually made in combination with other biomaterials because it is fluid at physiological temperature. Thus, its bioprintability and consistency are limited [50]. On the other hand, gelatin can be obtained from collagen by hydrolysis. Gelatin is a thermosensitive polymer also used in 3D bioprinting. Due to its origin, it is biocompatible and biodegradable. Like collagen, it is normally used in combination with other biomaterials. Gelatin can be made to react with methacrylic acid to form GelMA. This product is used in numerous tissue engineering applications due to its characteristics of photo-crosslinking, low immune response, biocompatibility, and printability [51,52].

Hydrogels have also been obtained from decellularized extracellular matrix as a material for bioinks. Decellularization, performed by chemical, physical, or enzymatic processes, allows the structure and composition of the extracellular matrix to be maintained. Therefore, it is a highly biocompatible biomaterial with important biomimetic properties. However, the mechanical properties of the decellularized extracellular matrix are very weak. Thus, to improve its printability, it is subjected to different physical and chemical processes as well as combination with other biomaterials [53].

On the other hand, fibrin, derived from the enzymatic reaction between thrombin and fibrinogen, is also another interesting hydrogel for 3D bioprinting. It is mainly used for skin grafts and the treatment of scarring [54]. Additionally, silk, produced by different types of arthropods, is also a biomaterial that has recently been used in the field of biomedicine and bioengineering. Hydrogels based on silk or one of its most abundant proteins (fibroin) are highly biocompatible and biodegradable. They also have good combinability with other materials [55]. Likewise, the polysaccharide agarose, consisting of repeating units of β-D-galactose and 3,6-anhydro-L-galactopyranose, is used in the manufacture of bioinks due to its thermoreversible gelation. Thus, it has been used in combination with other biomaterials because its structure resembles the extracellular matrix. In addition, agarose allows oxygen and other products to diffuse through it, so its incorporation in bioinks helps support cellular functionality [34].

Hyaluronic acid is another component used in bioinks for its mechanical and adjustable properties. Among them, its high porosity and hydration capacity stand out. This favors the diffusion of nutrients and their use in maintaining a moist environment. That is useful, for instance, in wound healing [56]. Because cells cannot adhere to hyaluronic acid, in the manufacture of bioinks, it is used together with other biomaterials to increase cell adhesion [34]. This same situation occurs with dextran, which is a homopolysaccharide composed of a linear chain of D-glucoses linked by α-(1→6) bonds derived from lactic-acid bacteria. To be incorporated into bioinks, it must be chemically modified and conjugated with other cellular biomaterials [34].

Chitosan is a polysaccharide derived from chitin after a deacetylation process. Chitosan-based hydrogels have been used in numerous applications such as cartilage regeneration, vascular tissue, and the formation of wound dressings [57]. Unlike other natural materials, such as collagen, the degradation of chitosan is slower [58]. Another polysaccharide used as a source of components for the manufacture of bioinks is cellulose. This polymer, responsible for the structure of the plant cell wall, contains a high density of free OH groups that contribute to its insolubility. However, its chemical modification allows for the creation of soluble derivatives such as carboxymethylcellulose, methylcellulose, hydroxyethylmethylcellulose, hydroxyethylcellulose, and ethylcellulose. These can be incorporated into bioinks with applications in regenerative medicine [59]. Gums are also polysaccharides that are being used for the manufacture of bioinks due to their mechanical properties, biocompatibility, and biodegradation. Among them are gellan gum, produced by *Pseudomonas elodea*; konjac gum, derived from konjac tubers, xanthan gum, derived from *Xanthomonas campestris*, and guar gum obtained from seeds of *Cyamopsis tetragonolobus* [34].

Regarding synthetic biomaterials, they have the advantage of making it possible to control certain properties such as the degradation rate, mechanical strength, and structure. However, they may not be able to adequately mimic the composition of the extracellular matrix, and both their integration and degradation may produce cytotoxic compounds that affect their regenerative capacity [60]. Different types of synthetic materials have been used in tissue engineering, including metals, ceramics, composites, and polymers [61]. The first three have significant mechanical strength but polymers have better biocompatibility and biodegradation properties. Among these polymers are pluronic (copolymer based on polyethylene oxide (PEO) and hydrophobic polypropylene oxide (PPO)), polycaprolactone (PCL), poly(L-lactic) acid (PLA), polyvinylpyrrolidone (PVP), polyethylene glycol (PEG), and poly(lactic-co-glycolic) acid (PLGA). All of them are frequently used in the manufacture of bioinks for 3D bioprinting [40,61]. Currently, the manufacture of hybrid bioinks between natural and synthetic hydrogels has allowed the production of biomaterials for 3D bioprinting with high biocompatibility and printability [44].

In the last decade, new research on bioinks for 3D bioprinting has been rapidly developing. New compositions have been sought to satisfy different applications in tissue engineering and regenerative medicine. Interestingly, it has been found that mechanical and biological properties of biomaterials may be improved by the inclusion of nanoparticles (NP), thus forming nanocomposites. Depending on their chemical composition, nanoparticles can be based on (i) carbon nanoparticles, such as graphene oxide, graphene, and carbon nanotubes; (ii) ceramic nanoparticles based on silica, calcium phosphate, or bioactive glasses; (iii) biopolymeric nanoparticles of natural and synthetic origin; and (iv) metallic nanoparticles [62]. The inclusion of nanoparticles can also be used to deliver or release various bioactive compounds to promote cell growth and tissue regeneration. For example, an alginate-based bioink containing MSC and bone morphogenetic protein-2 (BMP-2) nanoparticles has been developed to print scaffolds with an enhanced osteoinductive capacity in bone regeneration [63]. In addition, NP can be used to deliver various bioactive agents to enhance cellular performance. For instance, several bioinks using alginate and PLGA NP encapsulated with BMP-2 to bioprint MSC-loaded scaffolds [63]. In addition to these combinatorial bioinks, new generations of biomaterials include living materials and transformable bioinks for 4D bioprinting. The former are composed of living cells involved in the synthesis or conformation of the bioink, while the latter have the ability to change shape or function over time after being printed. This incorporates time as another dimension in the manufacturing process and maturation of the structure [44].

## 5. Applications of 3D Bioprinting in Regenerative

Bioprinting is used mainly in four applications: (i) pharmacological research, with the aim of identifying and developing new drugs and studying aspects of pharmacokinetics; (ii) cell biology, to study biological processes of viability, proliferation, and intercellular connections; (iii) the study of tumor models, by recreating tumor microenviron-ments to investigate oncogenesis and potential therapies; and (iv) regenerative medicine, through manufacturing artificial tissues and organs, vascular development in tissues, and therapeutic processes of cell therapy [22].

Regenerative medicine is the main application of 3D bioprinting. Currently, numerous studies and developments of different bioinks and structures have been carried out in different fields of tissue regeneration such as bone [64,65,66], cartilage [67,68,69], nerve tissue [70,71], pancreas [72], liver [73], trachea [74,75,76], heart [77], endometrium [78,79], ovary [80], and skin [81,82] tissues. Table 1 shows a summary of the details of these studies aimed at the regeneration of different tissues using bioprinting technology. Although most of these studies are preclinical, it is possible to find registered trials for the evaluation of bioprinting techniques in tissue regeneration on web servers like ClinicalTrials of NIH <https://clinicaltrials.gov (accessed on 5 February 2025)>. For example, the one entitled “Patient-Customized Bioprinting Technology for Practical Regeneration of the Respiratory Tract (Trachea)” (ID NCT06051747).

## 6. Extracellular Vesicles and Regenerative Medicine

One of the mechanisms of intercellular communication is the secretion of extracellular vesicles (EV). They may contain proteins, nucleic acids, lipids, and other metabolites, which can affect different biological processes. It has been described how EV cargos can trigger functional responses and promote phenotypic and functional changes. This may affect recipient cells under physiological or pathological conditions [83]. Depending on the size and origin of EV, they can be classified as (i) microvesicles (20 to 1000 nm in diameter), formed from the evagination of the plasma membrane; (ii) exosomes, of endosomal origin (40 to 150 nm); and (iii) apoptotic bodies, derived from cells in the early stages of apoptosis (1000 to 5000 nm) [84]. However, the International Society for Extracellular Vesicles (ISEV), through the publication of “Minimal Information for Studies of Extracellular Vesicles” (MISEV), recommends the use of the term extracellular vesicles, defined as: “Particles that are released from cells, are delimited by a lipid bilayer, and cannot replicate on their own” [85].

EV can be released by almost all cell types, such as stem, epithelial, Schwann, inflammatory, neurons, endothelial, etc. [86]. In the body, EV can be isolated from most fluids, such as blood, urine, saliva, milk, and cerebrospinal fluid [87]. Also, from in vitro cell cultures, they can be isolated from conditioned culture media [88]. The most common methods to isolate them include ultracentrifugation, ultrafiltration, size exclusion chromatography, precipitation, and immunoaffinity purification or a combination of several. Once isolated, different methodologies can also be used for their characterization, including transmission electron microscopy (TEM), immunoblotting, flow cytometry, dynamic light scattering (DLS), and nanoparticle tracking analysis (NTA), among others [89].

The content of EV depends on the cell of origin and their physiological conditions. Interestingly, in this sense, recent studies have attributed the therapeutic effects of different types of stem cells to the extracellular vesicles they secrete [90]. Indeed, these vesicles are usually rich in factors that promote tissue regeneration and immunoregulatory cytokines. In addition, they do not exhibit immunogenic properties, which allow them to be applied exogenously to other individuals and even species, thus making them more practical from a clinical point of view [91]. Curiously, the preconditioning of MSC cultures with different types of stimuli such as hypoxia, inflammatory, mechanical, or pharmacological, affects the content of the EV. Therefore, these stimuli have been used as methods for obtaining EV enriched in certain factors and involved in certain regenerative processes. For example, EV derived from MSC grown in a hypoxic environment may have a greater angiogenic capacity, or those preconditioned with inflammatory factors may have greater immunomodulatory effects, as others and we have described [92,93].

In regenerative medicine, the use of EV is considered a cell-free therapy, presenting some important advantages with respect to cell therapy applications with MSC: (i) these vesicles are easy to isolate and store, without loss of potency of the load; (ii) the presence of lipid bilayers gives them stability, protecting against the degradation of their content; (iii) they can be applied in large doses, infiltrating different target organs; (iv) their administration can be intravenous, which will allow them to reach small capillaries; (v) they have the ability to cross the blood–brain barrier; and (vi) being a cell-free therapy, its application avoids possible tumorigenicity, cell dedifferentiation, embolism, or immune rejection [86]. In this way, current research strongly supports the potential of EV as a regenerative medicine intervention due to their biological components, which have shown efficacy on cancers as well as other skeletal, cardiac, and brain diseases [94].

**Figure 2:**
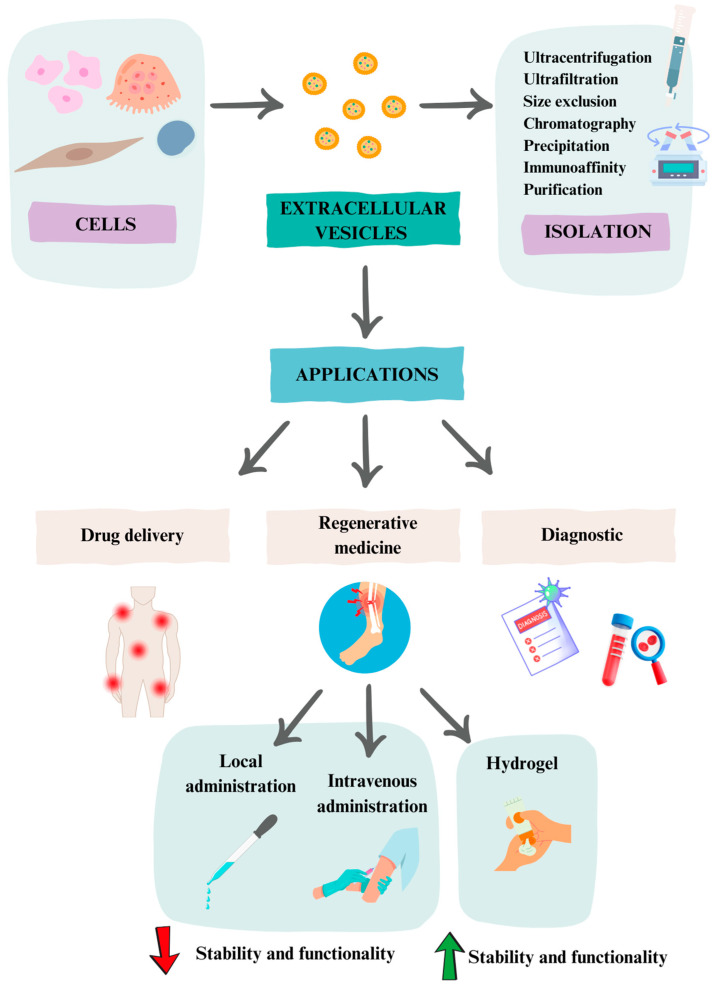
Application of extracellular vesicles (EV) derived from cell cultures in biomedicine. EV can be isolated from culture medium using different techniques. These EV have significant potential for clinical purposes, mainly in regenerative biomedicine. Their incorporation into hydrogels increases their stability and functionality.

Because the content of EV can reflect the physiological state of secretory cells, EV have recently become an important source of potential biomarkers for diagnostic and prognostic purposes. This has been further enhanced by the fact that they can be isolated from noninvasive liquid biopsies [95,96]. Also, from a therapeutic point of view, EV have a high potential to be used as a platform for drug delivery [97]. Thus, for example, preclinical studies have been conducted on the potential of EV to release nanoparticles and drugs for treatment of tumors, pulmonary, and neuronal pathologies, among other disorders (Figure 2) [98,99,100].

At the level of regenerative medicine, the development of cell-free strategies based on the application of extracellular vesicles is potentially very promising. However, direct application of EV directly into tissues is limited by their relatively rapid degradation. Thus, after intravenous injection of EV, most of the vesicles are eliminated in the liver and spleen and only 1% of EV can be detected 24 h after injection [101]. Also, local administration of EV presents limitations due to their rapid elimination in tissues. Therefore, to increase effectiveness, multiple doses may be necessary [102].

Fortunately, it has been recently found that hydrogels are effective at carrying and sustainably releasing extracellular vesicles into target tissues. Indeed, the incorporation of extracellular vesicles into hydrogels that can be used to form structures by 3D bioprinting represents a novel approach in tissue regeneration applications, such as wound healing [103]. This is because 3D bioprinting allows the precise arrangement of cells, biomaterials, EV, and growth factors in specific patterns and complex structures, achieving high reproducibility and functionality of the created structure. Hydrogels, through the maintenance of moisture and their lattice structure, can protect encapsulated EV from premature degradation, favoring their stability and functionality for a longer period of time (Figure 2). Furthermore, depending on the pore size and degradation rate, hydrogels can maintain a sustained release of embedded EV. This enhances the effectiveness of EV on tissues [103]. The encapsulation of EV in hydrogels can be performed in three ways: (i) By mixing the EV with the polymer and subsequently inducing gelation by addition or induction of crosslinkers; (ii) the “Breathing technique”, in which the hydrogel is first introduced into a solvent that removes water and then immersed in an aqueous solution containing the EV. This causes the insufflation of the exosomes into the hydrogel if the pore size of the hydrogel allows it; (iii) combining the polymer with the crosslinkers and the EV simultaneously, whereby the gelation of the hydrogel encapsulating the EV occurs at the same time [104]. Moreover, the binding of EV to the hydrogel in the encapsulation process can be facilitated by the affinity of certain integrins present on the surface of EV to extracellular matrix proteins. Thus, MSC-derived EV have been observed to bind to collagen and fibronectin peptides. An alginate-based hydrogel containing the RGD peptide of fibronectin allowed the encapsulation of these EV and their retention for 7 days, maintaining their integrity and functionality [105]. On the other hand, the release of EV from the hydrogel may be regulated in addition to the degradation rate of the biomaterial by other processes, such as changes in pH. This may affect the structure of the hydrogel, facilitating the release of EV [106].

The manufacture of bioinks that include EV may have the advantage of not needing to incorporate cells, taking into account that, in certain applications, EV have similar regenerative effects. This may facilitate the manufacture of bioinks and their 3D bioprinting due to greater flexibility in these processes as there is no need to maintain living cells. However, the encapsulation of EV in biomaterials does not exclude the use of cells in bioinks. Rather, it can be complementary to improve the viability and functionality of these cells and therefore the integration and regenerative effects of the bioprinted structures. The following sections will review the most recent advances in the use of 3D bioprinting of EV-loaded biomaterials in regenerative medicine, using both in vitro and in vivo assays.

## 7. In Vitro Evaluation of 3D-Bioprinted Hydrogels Loaded with Extracellular Vesicles

**Figure 3:**
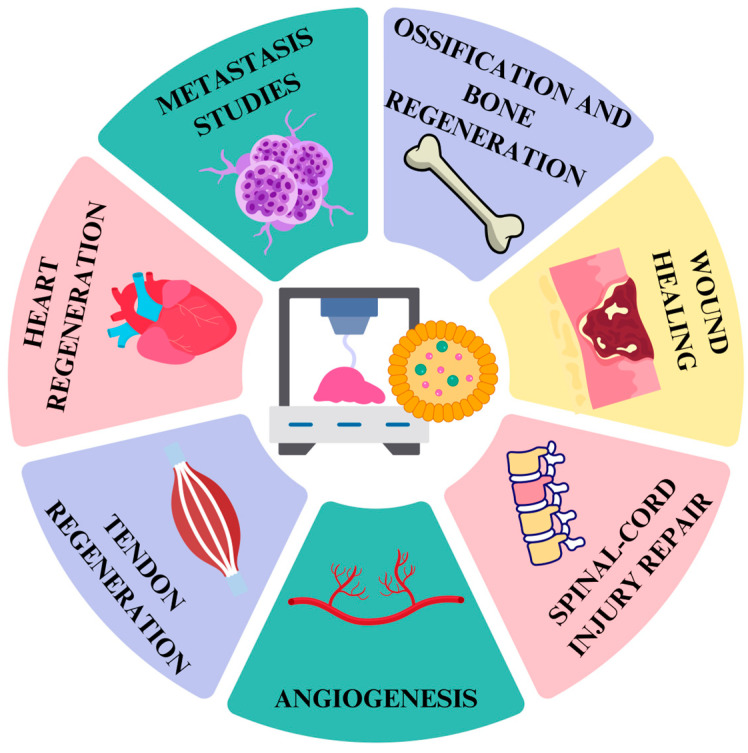
Applications of EV-loaded hydrogels in 3D bioprinting. EV can be incorporated into bioinks to fabricate scaffolds with diverse clinical applications, including wound healing, bone regeneration, cardiovascular therapy, and spinal-cord injury repair, among others.

As seen in the previous sections, stem cells are a powerful source of EV with a high regenerative capacity. They can be used in bioprinting, which is a promising therapeutic approach, avoiding potential immunogenic and tumorigenic effects of conventional cell therapies. This technique mimics the target tissue, using controlled pharmacokinetics and pharmacodynamics [107]. Numerous studies have evaluated the effects of structures obtained by bioprinting EV-loaded hydrogels in different cellular models. The objective of these studies was to ascertain the potential of these hydrogels to be applied in regenerative medicine, mainly aimed at wound healing, the induction of vascularization, and the regeneration of bone tissue, tendons, and heart tissue. They have also been used to build models for the study of tumor metastasis (Figure 3 and Table 2).

### 7.1. Wound Healing

MSC-derived EV have been shown to improve wound healing due to their immunomodulatory properties and great regenerative capacity. Thus, they can promote wound closure, increased re-epithelialization, cell proliferation, and angiogenesis [108]. Stem-cell-derived EV can be bioprinted with alginate/carboxymethyl cellulose hydrogels. This allows for the controlled EV release over time when it is in contact with a collagen-sheet-simulated skin. Such EV can even be lyophilized and stored to be bioprinted in extreme conditions [109]. Other authors have used different doses of MSC-derived EV to evaluate their performance on skin restoration. They demonstrated that 120 μg produced higher proliferation and tube formation in human umbilical vein endothelial cells (HUVEC). On the other hand, 40 μg promoted their migration, whereas 80 μg enhanced fibroblast migration and proliferation in comparison to controls. Then, they embed 20 or 120 ug of MSC-derived EV in methacrylated hyaluronic acid (MeHA or HAMA) wound dressings. The bioconstructs with 120 ug of EV promoted better EV release for up to 11 days [110]. On the other hand, collagen/skin-derived decellularized extracellular matrices with alginate, gelatin, and polydomamide, with or without M2 macrophage-derived EV, have also been recently tested. The coculture of fibroblast keratinocytes, MSC, and endothelial cells with bioprinted 3D hydrogels containing M2 EV enhanced cellular infiltration in the matrix as well as the proliferation and differentiation of cells after seven days. Additionally, genes like collagen type-I alpha 1 (*COL1A1)* were upregulated, promoting the maturation and differentiation of fibroblasts [111].

### 7.2. Angiogenesis

A challenge presented by bioprinting is the integration of a vascular system within the constructs to stimulate blood supply for the delivery of nutrients and oxygen [112]. A possible solution to that could be the addition of EV into the bioink. To evaluate this possibility, four different culturing conditions were used to obtain EV from HUVEC: normoxia, hypoxia, serum-free normoxia, and serum-free hypoxia for angiogenic applications. The HUVEC-derived EV were bioprinted in GelMA by microextrusion and cultivated with peripheral blood mononuclear cells (PBMC) containing endothelial progenitor cells (EPC) for seven days. This type of bioink is interesting since its crosslinking density can be easily adjusted, preserving both the integrity and mechanical strength of constructs [113]. Such a bioimprinted structure with EV derived from HUVECs grown in serum-free hypoxia improved the maturation of EPC. Additionally, it facilitated the development of a vascular-like network within the 3D-bioprinted constructs [114]. This demonstrates the potential of incorporating angiogenic EV in bioprinting to induce vessel formation.

### 7.3. Bone and Tendon Regeneration

EV-loaded bioinks have been applied in bone and tendon bioprinting, with promising results from studies in vitro. For instance, 3D-bioprinted EV constructs using 10 % GelMA were evaluated. EV derived from primary human periodontal ligament cells (HPDLC) and human gingival fibroblasts (HGF) encapsulated in this hydrogel were bioprinted using microextrusion methods. Then, human buccal fat pad-derived mesenchymal stromal cells (HBFP-MSC) were cultured on these constructs. In vitro assays such as cell attachment and osteogenic/cementogenic differentiation were carried out. The results showed successful EV release, improving cell adhesion and upregulation of gene expression profiles of the mechanotransduction pathway. GelMA/HPDLC-EV scaffolds increased the expression of genes encoding markers associated with ligament differentiation pathways (Mohawk homeobox (MKX), tenascin C (TNC), fibroblast-specific protein (FSP) and periodontal ligament-associated protein 1 (PLAP1)), bone formation (runt-related transcription factor 2 (RUNX2), bone sialoprotein (BSP), alkaline phosphatase (ALP)) and cement formation ((cementoblastoma-derived protein 1; CEMP1)). After two weeks with osteogenic and ligamentous differentiation, cells still had the capacity to differentiate [115].

Similar results were obtained using other types of EV and combining GelMA with silk methacrylate (SilMA). In general, silk can be used as a carrier for the delivery and sustained release of different products like enzymes, growth factors, etc., while maintaining EV biological activity [116]. Osteogenic proliferation and differentiation were studied in such constructs. GelMA/SilMA (G/S) bioinks were loaded with Schwann cell (SC)-derived EV (SC-EV) and 10^7^ cells/mL of bone marrow stem cells (BMSC), being cultured for seven days. Compared to the controls (G/S with BMSC), the presence of EV offered higher osteogenic activity, increasing the expression of *RUNX2* and osteopontin (*OPN*). This shows that SC-EV could improve the bone regeneration microenvironment [117].

In this context, similar studies using alternative constructs have been carried out to analyze the osteogenic and angiogenic abilities of MCS. Thus, hydrogels were constructed by combining a decellularized extracellular matrix (dECM), gelatin (Gel), chitosan (QCS), and nanohydroxyapatite (nHAp) particles loaded with EV from human-adipose-derived stem cells to obtain a 3D-printed hybrid scaffold. These constructs offered suitable structures, stimulating MSC proliferation and osteogenic differentiation. In fact, the presence of EV promoted the angiogenesis-associated gene expression of vascular endothelial growth factor (VEGF) and cluster of differentiation 31 (CD31) as well as the enhanced expression of ALP and RUNX2 osteogenesis markers [118].

On the other hand, scaffolds made from collagen-coated coverslips were used to study their osteogenic bioactivity. Thus, EV from the murine J774A.1 monocytic cell line were modified, introducing bone morphogenetic-protein 2 (BMP2) by electroporation and sonication. This protein is involved in bone formation and regeneration [119]. Using C2C12 myoblast cells cultures, an enhancement in osteogenic ALP activity was observed after three days in a culture in the presence of the scaffolds containing EV loaded with BMP2 [120]. Another study showed that bioprinted scaffolds loaded with EV offered a promising approach for tendon tissue engineering. Thus, stem-cell-derived EV can enhance scaffold biomechanical and biochemical properties, creating optimal microenvironments for cell differentiation [121]. In this scenario, it has been demonstrated that EV-loaded PCL and type-I collagen (COL1)-based scaffolds in culture with rabbit bone marrow stem cells significantly promoted tenocyte proliferation. Additionally, high induction of genes and synthesis of proteins associated with tendon regeneration, like tenascin-C and COL1A1, were observed [122].

### 7.4. Heart Regeneration

It has been shown that certain microribonucleic acids (microRNA or miRNA) can promote cardiac recovery by inducing antiapoptotic effects, promoting matrix proliferation, and regulating inflammatory responses. Likewise, they have also been related to the attenuation of fibrosis [123]. In particular, mir-199a-3p activates the transcriptional cofactor yes-associated protein (YAP), promoting cardiac regeneration after myocardial injury. Thus, miR-199a-3p mimic was loaded into EV from human monocytic cell line THP-1-derived activated macrophages (MΦ). Then, they were included in a bioink based on alginate-Arg-Gly-Asp (alginate-RGD), gelatin, and rat neonatal cardiomyocytes (NRCM) to form cardiac patches. Interestingly, EV showed a controlled release of miR-199a-3p within the patches, which was beneficial to maintain its cardioprotective effects. Also, increased cardiac cell survival, decreasing significantly markers of cell damage and apoptosis [124].

On the other hand, EV can be combined with liposomes, generating hybrid structures that may improve stability and loading of microRNA. Thus, cardiac patches were manufactured using 7.5% GelMA matrices with interspersed cardiac cells and nanovesicles. Compared to the control groups (liposomes or EV alone in the cardiac patches), hybrid bioinks improved cell viability by promoting environments that increased cell survival and proliferation. In addition, they allowed the sustained releases of microRNA, promoting cell differentiation [125].

### 7.5. Metastasis Studies

Three-dimensional printing can also be used to create microenvironments and structures of both healthy and diseased organs [126]. In such a scenario, they can represent interesting methods to study metastases, reprogramming cells into other phenotypes. Thus, liver-on-a-chip (LOC) designs have been proposed using bioprinting GelMA hydrogels loaded with hepatocyte extracellular vesicles in a microfluidic chip. Additionally, reprogrammed triple-negative breast cancers were bioprinted with bioinks loaded with EV. Then, reprogramming processes took place to produce hepatocytes via the lentiviral expression of genes encoding hepatocyte nuclear-factor 4 (HNF4), forkhead box proteins A2 and A3 (FOXA2 and FOXA3, respectively), activating transcription-factor 5 (ATF5), prospero homeobox-protein 1 (PROX1), and hepatocyte nuclear-factor 1 (HNF1). This provided a mechanism to improve cell microenvironments, promoting changes toward the hepatocyte phenotype [127].

### 7.6. Plant-Derived EV in Bioprinting

It has recently been demonstrated that plant-derived EV (PEV) may have significant therapeutic potential in regenerative medicine [128]. Therefore, they have also been used as components of structures made by bioprinting for therapeutic purposes. Indeed, a combination between plant-derived EV with a therapeutic compound of isoliquiritigenin in 3D-printed scaffolds is promising for improving spinal cord injury (SCI) repair. In such scaffolds, the incorporation of EV from Chinese wolfberry (*Lycium barbarum)* significantly improved neuronal differentiation in comparison to ectomesenchymal stem-cell-derived exosomes. Markers such as cyclooxygenase 2 (COX2), inducible nitric oxide synthase (iNOS), tumor necrosis factor alpha (TNF alpha), and interleukin-6 (IL6) were inhibited, so it also revealed anti-inflammatory properties, reducing activation of proinflammatory cytokines [129].

**Table 2:** Applications of extracellular-vesicle-loaded 3D bioprinting scaffolds in in vitro studies.

Treated Cells	EV Origin	EV Isolation	Bioink	Bioprinting Technique	[EV] Bioink	Key Findings	Ref.
Angiogenesis							
PBMC	HUVEC	Ultracentrifugation from normoxia and hipoxia medium	GelMA	Extrusion bioprinter	4 × 10^9^ particles/mL	EV generated under hypoxic conditions elicited a stronger angiogenic stimulus	[114]
Wound healing							
	MSC	Ultrafiltration from the conditioned medium	HAMA		20 ug 120 ug	EV release was proportional to loaded doses	[110]
Human skin cells	RAW 264.7	Exo kit	Epidermal, dermal, lumen, and neural	Multilayered		Exo-treated model showed excellent cellular infiltration and proliferation	[111]
Bone and tendon regeneration							
hBFP-MSC	hPDLC hGF	Conditioned media using size exclusion chromatography	GelMA	Extrusion bioprinter	10 ^10^ particles/mL^−1^	Significantly promoted MSC proliferation, migration, and osteogenic differentiation	[115]
BMSC	SC	Gradient low-temperature ultracentrifugation	GelMA-SF	Extrusion bioprinter	10^7^ cells/mL^−1^	Higher osteogenic activity	[117]
hBMSC	hASC	Differential ultracentrifugation	dGQH	Extrusion bioprinter	30 μg/mL	Enhanced the expression of osteogenesis markers	[118]
C2C12	J774A.1 monocytic cell line		ADM	Printed onto collagen type I-coated coverslips	100 μg/mL	Induced localized osteogenesis	[120]
Rabbit BMSC	TSC	Ultracentrifuged TSC-conditioned medium	PCL-Col	Form crossed strands	1 × 10^11^ particles/mL	Enhanced tenocytes proliferation	[122]
Heart regeneration							
Rat cardiomyocytes	THP-1-derived activated macrophages	Culture media using differential centrifugation and ultrafiltration	Alginate-RGD gelatin	Extrusion bioprinter	1.2 × 10^11^ EV/mL	Apoptotic markers were lower in cardiac patches with EV	[124]
Neonatal rat cardiac fibroblasts and myocytes	Neonatal rat cardiac fibroblasts and myocytes	Aqueous two-phases system	GelMA		100 μg/mL	Promote cell proliferation	[130]
Metastasis studies							
TNBC	Hepatocytes	Ultracentrifugation	GelMA	Microfluidic chips	20 μg/mL 60 μg/mL 200 μg/mL		[127]

ADM: acellular dermal matrix; BMSC: bone marrow mesenchymal stem cell; BMP-2: Bone Morphogenetic Protein-2; dECM: decellularized extracellular matrix; dGQH: decellularized extracellular matrix (dECM), gelatin (Gel), quaterinized chitosan (QCS) and nanohydroxyapatite (nHAp); EV: extracellular vesicle; Exo: exosome; GelMA: methacrylated gelatin; HAMA: methacrylated hyaluronic acid; hASC: human adipose-derived stem cell; hBFP-MSCs: human buccal fat pad-derived mesenchymal stromal cells; hBMSC: human bone marrow mesenchymal stem cell; hGF: human gingival fibroblasts; HUVEC: human umbilical vein endothelial cell; MSC: mesenchymal stem cell; PBMC: peripheral blood mononuclear cell; PCL-Col: polycaprolactone (PCL) microfibers loaded with cholecalciferol (Col); SC: Schwann cell; SF: silk fibroin; TSC: tendon stem cell.

## 8. Preclinical Applications of 3D-Bioprinted Hydrogels Loaded with Extracellular Vesicles

The development in recent years of the incorporation of EV into hydrogels for 3D bioprinting has given rise to various preclinical in vivo investigations. Progress in this type of study is essential to evaluate the potential clinical use in regenerative medicine. Specifically, studies have been conducted on the effect of these products on revascularization, tendon and bone regeneration, spinal cord injury repair, and wound healing (Table 3).

### 8.1. Angiogenesis

Different studies have proposed the use of EV as bioadditives for 3D-bioprinted scaffolds, to promote tissue neovascularization. Angiogenic properties have been evaluated in nonobese diabetic (NOD) scid gamma (NSG) mice transplanted with 3D-bioprinted thermosensitive GelMA scaffolds containing HUVEC-derived EV. These scaffolds were implanted in dorsal, cranial, and caudal surgical sites for 60 days, generating a massive presence of functional blood vessels on the muscle surface. Furthermore, histological analyses revealed the presence of well-organized vascular networks. Such neovasculature self-assembled both outside and inside the 3D-bioprinted scaffolds, showing the ability of EV to attract endothelial precursor cells [114].

EV isolated from other sources of stem cells have exhibited similar results. For instance, a hybrid scaffold was used loaded with EV isolated from human-adipose-derived stem cells. This was carried out on a critical-size (6 mm) rat calvarial defect model. After one week, the collected samples reflected the angiogenic potential of the EV-loaded 3D-bioprinted scaffolds. Thus, they showed a higher number of HUVEC and upregulated expression of genes encoding angiogenic markers (CD31 and VEGF) [118]. The increase in angiogenesis and vascular cells may be of great relevance in tissue regeneration. Indeed, they affect biological processes, such as bone regeneration, at several different levels [131]. In the same way, 3D-bioprinted scaffolds loaded with Schwann-cell-derived EV have demonstrated an increase in angiogenesis in a cranial defect model [117]. These results indicate that SC-derived EV-loaded 3D-bioprinted scaffolds could also improve cell microenvironments by promoting angiogenesis and other processes critical for tissue regeneration.

In the field of otorhinolaryngology, 3D bioprinting is a tool with high therapeutic potential. Thus, artificial tracheas have been fabricated by 3D impression with sericin microsphere and coincubation with tracheal epithelial cells and chondrocytes. This microtissue allows cell adhesion and proliferation and can be used for the treatment of tracheal defects [132]. The use of this technology has also been suggested for the repair of the tympanic membrane and preclinical studies have been performed with different models [133]. However, so far, very few studies have used 3D bioprinting of EV-loaded hydrogels in otolaryngology-related applications. Recently, with the aim of promoting angiogenesis in 3D-printed PCL/GelMA fabricated scaffolds, endothelial progenitor cell-derived EV were incorporated and they promoted tracheal regeneration in rabbits. This effect was as a consequence of the induction of tracheal cell adhesion and proliferation as well as vascularization of the implant [134].

As has been made clear in most of these studies, for applications in which it is desirable to promote angiogenesis, GelMA biomaterial exhibits favorable biological properties that promote cell adhesion and proliferation along with tunable physicochemical characteristics influenced by prepolymer concentration, methacrylation degree, and photopolymerization parameters. Moreover, it is cost effective, nonimmunogenic, and highly biocompatible. Additionally, it provides structural support and enhances resistance to enzymatic degradation, making it a promising candidate for biomedical applications [135]. Furthermore, its organized structure acts as a guide to facilitate vascularization. However, GelMA hydrogels can be combined with other types of materials such as silk fibroin (SF) that acts as an excellent carrier for EV that allow their gradual release [117].

### 8.2. Tendon Regeneration

EV have also been proposed to achieve therapeutic effects in tendinous tissues healing and regeneration. Loading EV into scaffolds may be an effective practical strategy for tissue regeneration without loss of bioactivity [136]. Previous studies implanted tendon-stem-cell-derived EV-loaded PCL scaffolds in male New Zealand rabbits [122]. Regenerated tissues were abundant and organized along the longitudinal axis of the tensile force. Furthermore, the tendon-to-bone insertion site was continuous at week eight. After 16 weeks, parallel fibers with large diameters (with same characteristics as mature tendon fibers) were more abundant within the tendon-stem-cell-derived EV-loaded scaffold. In addition, regenerated tissues showed signs of internal maturation. Successful regeneration and remodeling of tendon tissues is characterized by a switch from collagen type 3 to type [137,138,139]. Additionally, the maintenance of collagen type 3 formation represents adhesion or fibrovascularization rather than regeneration [138,140]. In this scenario, EV-loaded-scaffolds showed positively and negatively stained areas of type 1 and 3 collagen, as revealed by immunohistochemical staining at week eight 8 and 16. Ultimate load, stress to failure, and stiffness increased also from week 8 to 16. The effects of this novel therapy could be attributed to the activation of energy metabolism pathways and immunoregulation in the early postoperative stage, creating a better environment for healing [141].

PCL is commonly used in tissue repair due to its excellent biocompatibility and low immunogenicity [142]. PCL is a promising biomaterial because it has a degradation rate of two to four years, offering the possibility of being replaced by functional tissue by the body itself. In addition, its elasticity depends on the molecular weight and it has greater rigidity than other polymers such as PGA [143]. Usually, it is combined with type 1 collagen, an extracellular matrix component, to enhance cell adhesion and differentiation [122].

Yet, other bioinks such as MeHA or GelMA have been proposed due to their mechanical properties and bioactivities. HAMA/GelMA double network (HG) hydrogels are promising strategies in tissue regeneration. HAMA and GelMA results in more porous and disorganized structures with a high swelling degree. In fact, strong covalent bonds are formed between them that make the structures difficult to break after an enzymatic attack [144]. These hybrid hydrogels could be loaded with bioactive factor and/or EV with different effects on regeneration. Previous studies have also shown how EV are effective carriers for the administration of bioactive factors involved in osteogenesis or osteochondral repair. One of these is the melatonin (MT), which has been related with proregeneration processes on cartilage due to its effects on inflammation, circadian rhythm, and synthesis of ECM [145,146]. Thus, a double network hydrogel, composed of HAMA and GelMA loaded with MT-EV was generated to create a cartilage layer. MT-EV played a relevant role promoting cartilage regeneration. They had the ability to polarize macrophage populations toward M2 type, which enhanced the catabolic activity of chondrocytes, thus promoting cartilage regeneration [146].

**Table 3:** Preclinical applications of 3D-bioprinted hydrogels loaded with extracellular vesicles.

Animal	EV Origin	Bioink	[EV] Bioink	Treatment Interval	Key Findings	Ref.
Angiogenesis						
NSG C57/BL6 mice	HUVEC	GelMA	4 × 10^9^ particles/mL	60 days	Increase in vascular network maturation and branching number	[114]
Sprague Dawley rats	Adipose-derived MSC	dGQH	30 μg/mL	10 weeks	Higher expression of angiogenic markers, enhanced vascularization after 10 weeks, blood vessel formation was increased at week one	[118]
Sprague Dawley rats	SC	GelMA-SF	1 × 10^7^ particles/mL	8 weeks	Increase in angiogenesis in a cranial defect model, higher number of vascular structures	[117]
Rabbit	EPC	PCL/GelMA	50 μg/mL	2 months	PCL/GelMA-EV scaffolds promoted vascularization, facilitating tracheal regeneration	[134]
Tendon regeneration						
New Zealand Rabbits	TSC	PCL-Col	1 × 10^11^ particles/mL	8 weeks 16 weeks	Tissues abundant and organized (internal maturation), tendon-to-bone insertion site was continuous at week 8, parallel fibers with large diameters, similar to mature tendon fibers at week 16, increase in type 1 and decrease in type 3 collagens, at weeks 8 and 16, respectively Ultimate load, stress-to-failure and stiffness increased at weeks 8 to 16	[122]
China white rabbits	Synovial MSC	HG		12 weeks	Promoted cartilage regeneration, polarized macrophage population toward M2 type	[146]
Osification and bone regeneration						
C57/BL6 mice	J774A.1 monocytic cell line	ADM	100 μg/mL	4 weeks	Localized heterotopic ossification in mouse muscle pocket model	[120]
Sprague Dawley rats	Adipose-derived MSC	dGQH	30 μg/mL	10 weeks	Collagenous bone cover and new bone tissue in defect areas, higher expression of osteogenic markers (ALP, RUNX2 and OCN)	[118]
Sprague Dawley rats	SC	GelMA-SF	1 × 10^7^ particles/mL	8 weeks	Increased *OCN* gene expression, new bone formation	[117]
Spinal-cord injury						
Sprague Dawley rats	MSC	GelMA LAP	5 mg/mL	8 weeks	Improved motor function compared to control groups, better regeneration of spinal nerves, with less edema and intercellular spaces, and higher number of spinal nerves, increased number of motor neurons in caudal ventral horn of spinal cord, increased neural markers	[129]
Wound healing						
Diabetic rats	MSC	HAMA	120 µg	7, 14, and 21 days	Less inflammation and quicker healing, modulatory effects on inflammatory factors and remodeling enzymes, tissue repair, as measured by reepithelialization	[110]

ADM: acellular dermal matrix; ALP: alkaline phosphatase; BMP2: bone morphogenetic protein 2; dECM: decellularized extracellular matrix; dGQH: decellularized extracellular matrix (dECM), EPC: endothelial progenitor cells, Gel: gelatin, QCS: quaterinized chitosan, nHAp: nanohydroxyapatite; EMSC: ectomesenchymal stem cell; EV: extracellular vesicle; GelMA: gelatine methacrylamide; HG: methacrylated hyaluronic acid and gelatin methacryloyl; HAMA: methacrylated hyaluronic acid; HUVEC: human umbilical-vein endothelial cell; ISL: isoliquiritin; LAP: lithium phenyl (2,4,6-trimethylbenzoyl) phosphinate; MSC: mesenchymal stem-cells; OCN: osteocalcin; PCL-Col: polycaprolactone (PCL) microfibers loaded with cholecalciferol (Col); SC: Schwann cell; SF: silk fibroin; TSC: tendon stem cell; VEGF: vascular endothelial growth factor.

### 8.3. Ossification and Bone Regeneration

In the scientific literature, some studies use GelMA as the base of hybrid hydrogels combined with other bioinks such as SilMA for bone regeneration. In line with the angiogenesis materials, GelMA has also been used as a bone repair material due to its good temperature-sensitive gel properties, degradability, adjustable mechanical properties, and ability to promote bone differentiation and vascularization [147]. When it is combined with silk fibroin, its osteogenic properties improve, providing greater flexibility, adjustable mechanical properties, biocompatibility, osseointegration, and a better permeability to water and oxygen [148,149,150], making bioink the most chosen for bone tissue regeneration processes. It can be used as a carrier of EV, maintaining the biological activity of such vesicles for a longer time in addition to releasing them slowly [151,152]. The in vivo bone regeneration capacity of a hybrid scaffold loaded with EV isolated from human adipose-derived stem cells was investigated in the rat skull defect model by implantation for 10 weeks. The group treated with this scaffold presented a collagenous bone cover and new bone tissue in the defect area compared to the control one, which presented mainly fibrous tissue. The results of immunohistochemical staining for ALP, RUNX2, and osteocalcin (OCN) showed the highest expression values of these osteogenic markers [118]. In this study, a hydrogel based on a combination of ECM, gelatin, QCS, and nHAp was used. It is characterized by its high porosity, antibacterial activity, hemocompatibility, and biocompatibility. Gelatin promotes heat-resistant behavior and favors cell adhesion and migration; QCS gives it good solubility in water with antibacterial activity and nHAp improves osteoconductivity, bioactivity, and mechanical resistance in addition to reducing scaffold degradation [118].

On the other hand, nerve–bone communications are essential for bone repair. Thus, Schwann cells are crucial in regulating cell microenvironments through EV secretion [153]. Therefore, some studies have proposed the use of SC-derived EV as a therapy for bone tissue regeneration. Indeed, Sprague Dawley (SD) rat tissues treated with 3D-bioprinted scaffolds loaded with such EV exhibited increased OCN expression, confirming nerve–bone-mediated communication to regulate osteogenic differentiation. Furthermore, increased innervation and a higher number of vascular structures as well as increased new bone formation in the cranial defect model used were observed [117]. These results indicate that 3D-bioprinted scaffolds loaded with SC-derived EV could improve cellular microenvironments by releasing EV with the ability to exert reparative effects, promoting innervation and angiogenesis and thus effective bone repair.

Recombinant human BMP2 is considered a paradigmatic growth factor due to its biological and clinical importance. The use of EV delivery vehicles for BMP2 could be interesting due to its high capacity to bind to extracellular-matrix components and to protect intraluminal cargos from extravesicular degradation by antagonists/inhibitors/enzymes [154]. Therefore, 3D bioprinting was used to create a collagen-rich acellular-dermal matrix (ADM) material that also contained BMP2, directly loaded into lumens of engineered BMP2 EV (eBMP2-EV). Such EV were implanted into male C57BL/6 mice muscle pockets. Analyses of X-ray microcomputed tomography (μCT) radiographic images as well as hematoxylin and eosin (H&E) and Masson’s trichrome staining showed spatially controlled heterotopic ossification formation at the scaffold implantation site [120]. In this case, the use of ADM is proposed to be an excellent biomaterial for bone regeneration. It allows cell proliferation, avoiding inflammatory responses. Also, it maintains tensile properties that can simplify the implantation and manipulation [120].

### 8.4. Spinal Cord Injury Repair

Implementing 3D-bioprinted scaffolds could also facilitate SCI repair by controlling drug release, promoting synaptic regeneration, and reducing scar formation [155]. In the treatment of SCI, isoliquiritin (ISL), a flavonoid derived from licorice, may have anti-inflammatory and neuroprotective effects [156]. They could be enhanced by encapsulation in PEV, improving their solubility and pharmacological activity. SD rats were implanted with a GelMA hydrogel scaffold containing ISL encapsulated within PEV from *L. barbarum* (ISL@PEV) with the aim to promote SCI repair. Motor functions, evaluated by inclined plane tests, Basso, Beattie, and Bresnahan (BBB) scores as well as open-field tests, were improved compared to the control groups. In addition, only rats in the ISL@PEV-treated group could support their weight while standing and walking. Histological analyses also showed that treatment with ISL@PEV resulted in better regeneration of spinal nerves with less edema and intercellular spaces as well as a higher number of spinal nerves. In addition, motor neurons in the caudal ventral horn of the spinal cord increased in the hydrogel groups compared to the control ones. Additionally, a significant increase in neural markers was observed in the ISL@PE-treated group. Therefore, the authors conclude that the implantation of a 3D-bioprinted scaffold containing such drug encapsulated in EV can promote the repair and regeneration of damaged neuronal cells, thus achieving spinal cord repair due to the synergic effects of ISL and PEV [129]. In this application, it should be noted that for the use of GelMA-based hydrogels, its elasticity and Young’s modulus must be close to that of the soft extracellular matrix of neural tissue and it must also have a greater resistance to fatigue and more water content [157].

### 8.5. Wound Healing

Three-dimensional bioprinting has recently been suggested as an attractive option in wound healing [158]. In this regard, HAMA, due to its intrinsic rheological characteristics, is considered an ideal bioink as it is also biodegradable, lacks immunogenicity, and can be easily loaded with bioactive molecules such as small EV [130,159,160]. It is composed of hyaluronic acid, which exhibits high biocompatibility, a high capacity to maintain moisture, and anti-inflammatory effects, and methacrylate, which is applied to enhance mechanical stiffness, long-term stability, and to ensure a more controlled release of EV [110]. Several studies have shown promising results in the treatment of chronic wounds by using hydrogels as carriers for the controlled delivery of small EV from human MSC [161,162]. In a diabetic mouse model of pressure ulcer, wounds treated with 3D-bioprinted HAMA scaffolds containing MSC-EV and surrounding skin appeared visually less inflamed and also healed more quickly than wounds treated with standard care. In addition, such treatments had a modulatory effect on inflammatory factors interleukin-1 beta (IL1b) and monocyte chemoattractant-protein-1 (MCP1) and remodeling enzymes, including matrix metallopeptidase 2 (MMP2) and matrix metallopeptidase 9 (MMP9), involved in wound healing. Tissue repair, as measured by re-epithelialization, angiogenesis, and terminal innervation, was enhanced in mice whose HAMA scaffolds carried MSC-EV [110]. This fact may be of great relevance in the healing of purely neuropathic and neuroischemic diabetic foot ulcers [163].

One of the challenges of this therapy in wound healing is the mechanical properties of hydrogels, which could be improved by incorporating new biopolymers or mixing them with inorganic materials. Additionally, it is important to consider storage conditions and how long they remain biologically active in EV [164]. In fact, a possibility could be to lyophilize EV and make them active when applying the hydrogel [109]. In the treatment of chronic skin ulcers that require continuous care over long periods of time, the possibility of using dressings made by bioprinting EV-loaded hydrogels would facilitate their application in primary health care.

## 9. Conclusions and Perspectives

In the last decade, the development of new bioinks, in parallel to new 3D bioprinting technologies, has brought about new milestones in the advancement of tissue engineering and regenerative medicine. A multitude of studies have shown the potential of this technology in the artificial fabrication of structures, replacing damaged tissues or organs, or inducing their regeneration [35]. The goal in the next decades is to make 3D bioprinting a means of on-demand manufacturing of tissues or organs, thus revolutionizing organ transplantation. Yet, there are still many challenges at the level of research and technological development to be overcome. Among the most important ones is the development of suitable biomaterials. These should exhibit biocompatibility and the ability to support cell growth and differentiation. Additionally, they must have the appropriate mechanical properties to support complex and bulky structures. Nevertheless, such goals have not been completely reached on many hydrogels used in the manufacture of bioinks. It must also be taken into account that, in order to design a structure as complex as a transplantable artificial organ, there are numerous factors that affect this. They include the selection of biomaterials, usage of different cell types in the same structures, development of vascular and innervation tissues that guarantee their viability as well as possible immune responses and their regenerative potentials, among others [16]. All of this implies that multidisciplinary studies need to be carried out, where engineering, chemistry, biotechnology, and medicine converge in a coordinated manner in such a multidisciplinary topic. All this, together with the need to standardize the methods used and regulate the use of these complex technologies through consensus promoted by public drug agencies, constitute challenges that must be met in order for the clinical use of 3D bioprinting to become a reality [165,166].

The incorporation of EV in bioinks is another advance in bioprinting processes, optimizing regenerative capacities of created structures. On the one hand, due to the regenerative capacity of EV, their use can address the drawbacks that may have the inclusion of living cells in bioink. Thus, studies on encapsulation of EV in hydrogels used in 3D bioprinting have been carried out. They have demonstrated the high therapeutic potential of this strategy in regenerative medicine. Most of such works have evaluated the incorporation of EV into these structures without adding cells in the bioink. However, in the fabrication of complex structures, it will probably be necessary to incorporate different cell types during 3D bioprinting. The rationale is to enhance the functionality of such artificial tissues and organs. In these cases, the presence of EV in bioinks can also play an important role in creating appropriate microenvironments. That way, they could improve both viability and functionality of such cells. This can be accomplished during bioprinting as well as while integrating such structures into injured tissues and organs.

Incorporating EV in tissue engineering through bioprinting must go together with other considerations. These include the optimization and standardization of methods for obtaining and purifying EV. In addition, it is necessary to ascertain which EV origin would be the most suitable for each application. In this context, culture conditions of secretory cells must also be taken into account. The rationale is that preconditioning cells with different stimuli may affect EV contents. This may significantly influence the functionalities of EV. Those are therefore factors to be taken into account, depending on their applications [92]. Thus, depending on the content of the EV, their effects may differ according to the recipient cell type. Therefore, a homogeneous distribution of EV incorporated into scaffolds may not exert the desired effect. To correct this, one advantage of bioprinting is that it allows EV from different origins to be included in the same structure. This way, each can be distributed in specific areas according to their functions. This may have great applicability, mainly for manufacturing complex tissues or organs. This is because in such scenarios, different cell types and extracellular matrices may not be homogeneously distributed.

The future development of 3D bioprinting applied to regenerative medicine must go together with the establishment of ethical parameters and good manufacturing practices (GMP) to ensure the biosafety of therapeutic procedures. This is because the inclusion of living cells together with biomaterials in the 3D printing process has ethical and regulatory implications. These affect the sourcing of cells and materials, biocompatibility of the fabricated structure, degradation of the printed materials, clinical application, and long-term treatment effects. Regarding the cell type used, whether for incorporation into the bioink or for obtaining EV, it is necessary to consider whether they are of embryonic origin, induced pluripotent stem cells (iPSCs) or adult-derived stem cells. The use of the former is subject to significant ethical controversy and is therefore regulated by strict norms in most developed countries. In many of these countries, only the procurement of embryonic cells from excess embryos derived from assisted reproduction techniques is allowed. As for the rest of the cell types, their use is less ethically problematic, although in the case of donations, it is always necessary to have informed consent and procedures to maintain anonymity. Independently of cell origin, it is necessary to guarantee through the corresponding preclinical and clinical studies that there are no immunological, disease transmission, or tumor development risks, among other factors. As for the biomaterials used, as with cells, it is important to take into account ethical aspects regarding their origin, environmental impact, and biocompatibility. As mentioned in this review, biomaterials and the metabolites derived from their degradation must not be toxic, immunogenic, or have negative effects on health in the long term. Thus, their composition and manufacture must be regulated at the legislative level [167]. Therefore, with respect to the ethical and biosafety aspects that the development of therapies based on complex technologies such as 3D bioprinting of biomaterials loaded with cells and/or other bioactive compounds such as EV must comply with, one of the main challenges is the creation of consensus guidelines on the entire manufacturing process. To this end, it is essential to take into account the guidelines approved by various drug regulatory agencies such as FDA (Food and Drug Administration; USA), the State Food and Drug Administration (SFDA; China), or the European Medicines Agency (EMA; Europe), among others, in addition to fulfilling GMP and the International Organization of Standards (ISO) standards for the regulation of medical devices and the manufacture of tissue engineered products [168].

In conclusion, there are still some challenges to be addressed in clinical practice for the general use of 3D bioprinting as a tool in tissue regeneration. Nevertheless, the advances being made in recent years show the great potential of this technology. In this way, incorporating EV as biological components in bioinks is an important step. Indeed, this improves the regenerative capacities and functionalities of structures manufactured by 3D bioprinting. The high level of interest in this technology in recent years is evidenced by the large number of patents registered in relation to this strategy. In particular, we have detected 84 related to this topic in a search carried out in March 2025 at the European Patent Office (see Supplementary Material). However, it is still necessary to make further progress in demonstrating the therapeutic capacity observed in the preclinical assays performed to date. To this end, it is essential to advance in the design and development of clinical trials to demonstrate the therapeutic application of this technology.

## Figures and Tables

**Table 1:** Main applications of bioprinting in regenerative medicine.

Animal	Tissue	Cell Source	Bioink	Bioprinting Technique	Key Findings	Ref.
Rat	Bone	Rat osteoblasts	PEGDA Laponite	Extrusion bioprinter	Promotion of cell graft, survival, and, therefore, long-term bone regeneration	[64]
Rat	Bone	rBMSC	Alginate Gelatin	Extrusion bioprinter	Acceleration of regeneration, increased cell adhesion, and osteogenic differentiation	[65]
Mice Rat	Bone	rBMSC	PLCL/nHA	Extrusion bioprinter	Promote tissue regeneration and vascularized	[66]
Mouse	Cartilage	pMSC	Gelatin Alginate Alginate sulfate	Extrusion bioprinter	Improves chondrogenesis and the controlled release of TGF-β3, promoting the cartilage-specific extracellular matrix	[67]
Mouse	Cartilage	Rabbit MSC	dECM-SF	Extrusion bioprinter	Increases the expression of chondrogenesis-specific genes and release of TGF-β3, promoting chondrogenic differentiation and cartilage repair	[68]
Rabbit	Cartilage	Synovial MSC	PCL	Extrusion bioprinter	Acceleration of regeneration, cell proliferation, and chondrogenesis and slowed the development of osteoarthritis	[69]
Mouse	Neural	Rat Schwann cells	Gelatin and Alginate	Extrusion bioprinter	Improved cell adhesion and expression of factors related to neural tissue regeneration	[70]
Mice	Neural	Neural crest stem cell-derived Schwann cells	PCL	Extrusion bioprinter	Promotion of peripheral nerve regeneration with improved anatomy	[71]
Mice	Pancreas	ASC	NFC	Extrusion bioprinter	Promising effects on islet viability, glucose sensing, insulin secretion, and reduction in proinflammatory cytokine secretion	[72]
Rat	Liver	HUVECs hMSC	GelMA Fibrin	Extrusion bioprinter	Recapitulation of the vascular network and maintenance of optimal tissue cellularization	[73]
Rat	Trachea	Human dermal fibroblasts Human articular chondrocytes HUVECs hMSC	Multicellular spheroids	Inject printing	Proliferation of the tracheal epithelium and capillaries	[74]
Mice	Trachea	Rabbit auricular chondrocytes Skin dermis fibroblasts	GelMA CSMA ACMMA	Extrusion bioprinter	Functional reconstruction of the trachea was achieved, both in its mechanical and physiological characteristics	[75]
			HAMA 8-PEG NHS ADMMA	Photo crosslinking		
Mice	Trachea	Human chondrocytes	PLCL/ Heparinized gelatin/ TGFb-1 gelatin Hydroxyapatite	Mixing extruded	Substantial cartilage regeneration capacity and appropriate mechanical behavior	[76]
Mice	Heart	hUC-MSC	Fibrin Gelatin aprotinin Glycerol Hyaluronic acid	Extrusion bioprinter	Promotes regeneration, increased survival of MSCs, reduced apoptosis, increased angiogenesis	[77]
Rat	Endometrium	Endometrial and epithelial stromal cells	Alginate hyaluronic acid	Extrusion bioprinter	Restoration of full-thickness morphology and fertility of injured uterine endometrium	[78]
Rat	Endometrium	Endometrial stromal cells	Gelatin alginate	Extrusion bioprinter	Improved endometrial regeneration	[79]
Mouse	Ovary	Ovarian cells	dECM-swine ovarian gelatin alginate	Extrusion bioprinter	Improvements in neoangiogenesis, cell proliferation, germ cell survival, and expression.	[80]
Rat	Skin	hGMSC	PCL	Extrusion bioprinter	Acceleration of wound closure with reduced scar formation	[81]
Mice	Skin	hADSCs	GelMA HAMA	Extrusion bioprinter	Accelerates wound healing and improves the quality of healing by promoting angiogenesis	[82]

8-PEG NHS: 8-arm-polyethylene glycol-succinic acid ester; ACMMA: methacryloyl-modified acellular cartilage matrix; ADMMA: methacryloyl-modified acellular derm matrix; ASC: adipose-derived stem cells; CSMA: methacrylate-modified chondroitin sulfate; dECM: decellularized extracellular matrix; GelMA: methacrylated gelatin; HAMA: methacrylated hyaluronic acid; hASC: human adipose-derived stem cell; hGMSC: human gingival mesenchymal stem cell; hUC-MSC: human umbilical cord mesenchymal stem cell; HUVEC: human umbilical vein endothelial cell; MSC: mesenchymal stem cell; NFC: alginate/nanofibrillated cellulose; PCL: polycaprolactone; PEGDA: polyethylene-glycol diacrylate; PLCL/heparinized gelatin/TGFb-1: copolymer poly (L-lactic acide-caprolactone)/ heparinized gelatin/ transforming growth factor-b1; PLCL/nHA: copolymer poly (L-lactic acide-caprolactone) hydroxyapatite nanoparticles; pMSC: porcine mesenchymal stem cell; rBMSC: rat bone marrow mesenchymal stem cell; SF: silk fibroin.

## Data Availability

No new data were created or analyzed in this study. Data sharing is not applicable to this article.

## Supplementary Materials

The following supporting information can be downloaded at: https://www.mdpi.com/article/10.3390/gels11030191/s1.

## Abbreviations

The following abbreviations are used in this manuscript:

ADM	acellular dermal matrix
ALP	alkaline phosphatase
BMP2	bone morphogenetic protein 2
dECM	decellularized extracellular matrix
dGQH	decellularized extracellular matrix
QCS	quaterinized chitosan
EMSC	ectomesenchymal stem cell
GelMA	gelatine methacrylamide
HG	methacrylated hyaluronic acid and gelatin methacryloyl
EV	extracellular vesicles
HUVEC	human umbilical-vein endothelial cell
ISL	isoliquiritin
LAP	lithium phenyl (2,4,6-trimethylbenzoyl) phosphinate
MSC	mesenchymal stem-cell
OCN	osteocalcin
PCL-Col	polycaprolactone (PCL) microfibers loaded with cholecalciferol
SC	Schwann cell
SF	silk fibroin
TSC	tendon stem cell
VEGF	vascular endothelial growth factor.
