# Supplementary material for: Bioprinted Hydrogels as Vehicles for the Application of Extracellular Vesicles in Regenerative Medicine
Source: Gels. 2025 Mar 8;11(3):191. doi: 10.3390/gels11030191 (PMC11941778; doi:10.3390/gels11030191)
Supplement: Supplementary file 1 [file gels-11-00191-s001.zip › gels-3508328-supplementary.pdf]

Espacenet

Result list

84 results found for exosomes 3d bioprinting hydrogel
Results 1 to 84 displayed
Query language: en / de / fr
Sort by: Publication date

---

1. DIGITAL ASSAY FOR SINGLE EXTRACELLULAR VESICLE, SINGLE MOLECULE DETECTION

Title	Inventors	Applicants	Publication number	Earliest priority	IPC	CPC	Publication date	Earliest publication	Family number
DIGITAL ASSAY FOR SINGLE EXTRACELLULAR VESICLE, SINGLE MOLECULE DETECTION	ROH YOON HO [US] MORALES RENEE-TYLER [US] KO JINA [US] REYNOLDS DAVID E [US] PAN MENGHAN [US]	UNIV PENNSYLVANIA [US] ROH YOON HO [US] MORALES RENEE TYLER [US]	WO2025024790A1	2023-07-26	G01N33/53<br>G01N33/574<br>G01N33/58<br>G01N33/68	G01N33/53 (EP)<br>G01N33/574 (EP)<br>G01N33/58 (EP)<br>G01N33/68 (EP)	2025-01-30	2025-01-30	094375453

2. PEPTIDE HYDROGELS, COMPOSITIONS, AND CONDITIONS FOR SCALABLE 3D CELL MANUFACTURING AND APPLICATIONS

Title	Inventors	Applicants	Publication number	Earliest priority	IPC	CPC	Publication date	Earliest publication	Family number
PEPTIDE HYDROGELS, COMPOSITIONS, AND CONDITIONS FOR SCALABLE 3D CELL MANUFACTURING AND APPLICATIONS	QI GUANGYAN [US] SUN XIUZHI [US]	UNIV KANSAS STATE [US] PEPGEL LLC [US]	WO2025015030A1	2023-07-10	B01D21/26<br>C12N5/02<br>C12N5/071<br>C12N5/0735	C12N5/0006 (EP)<br>C12N5/06 (EP)	2025-01-16	2025-01-16	094216404

3. 一种多尺度可控的三维组织构建的工艺方法及装置

Title	Inventors	Applicants	Publication number	Earliest priority	IPC	CPC	Publication date	Earliest publication	Family number
一种多尺度可控的三维组织构建的工艺方法及装置			CN119175879A	2024-09-02	B29C64/112<br>B29C64/20<br>B29C64/209<br>B29C64/245<br>B29C64/255<br>B29C64/329<br>B29C64/336<br>B29C64/393<br>B33Y10/00<br>B33Y30/00<br>B33Y40/00<br>B33Y50/02	B29C64/112 (CN)<br>B29C64/20 (CN)<br>B29C64/209 (CN)<br>B29C64/245 (CN)<br>B29C64/255 (CN)<br>B29C64/329 (CN)<br>B29C64/336 (CN)<br>B29C64/393 (CN)<br>B33Y10/00 (CN)<br>B33Y30/00 (CN)<br>B33Y40/00 (CN)<br>B33Y50/02 (CN)	2024-12-24	2024-12-24	093895835

4. HYDROGEL INK, HYDROGEL AND USES THEREOF

Title	Inventors	Applicants	Publication number	Earliest priority	IPC	CPC	Publication date	Earliest publication	Family number
HYDROGEL INK, HYDROGEL AND USES THEREOF	CHEW SING YIAN [SG] HUANG CHONGQUAN [SG] LAU KIERAN YAN KI [SG] LE THI NGUYET MINH [SG] JAYASINGHE MIGARA KAVISHKA [SG]	UNIV NANYANG TECH [SG] NAT UNIV SINGAPORE [SG]	WO2024237865A1	2023-05-18	A61K47/42<br>A61L27/52<br>A61P25/00<br>B33Y70/10	A61K47/42 (EP)<br>A61L27/18 (EP)<br>A61L27/20 (EP)<br>A61L27/222 (EP)<br>A61L27/52 (EP)<br>A61L27/54 (EP)<br>A61P25/00 (EP)<br>B33Y70/00 (EP)<br>A61L2300/252	2024-11-21	2024-11-21	093520136

						(EP) A61L2300/626 (EP) A61L2430/32 (EP) A61L27/18, C08L71/02, INV (EP) A61L27/20, C08L5/08, INV (EP)			

5. ANIMAL-FREE MATERIALS FOR CELL CULTIVATION IN BIOREACTORS AND METHODS OF MAKING AND USING THEREOF

Title	Inventors	Applicants	Publication number	Earliest priority	IPC	CPC	Publication date	Earliest publication	Family number
ANIMAL-FREE MATERIALS FOR CELL CULTIVATION IN BIOREACTORS AND METHODS OF MAKING AND USING THEREOF	AHADIAN SAMAD [US] GRAY STEPHEN G [IE]	NOUBIO INC [US]	US2024352411A1	2023-04-20	A23J3/22 C12N5/00	A23J3/227 (EP,US) C12N5/0075 (EP,US) C12N2531/00 (EP,US) C12N2533/50 (EP,US) C12N2533/70 (EP,US) C12N2533/74 (EP)	2024-10-24	2024-10-24	093122023

6. POLYMER MATERIAL CONTAINING CHEMICALLY MODIFIED POLYSACCHARIDE

Title	Inventors	Applicants	Publication number	Earliest priority	IPC	CPC	Publication date	Earliest publication	Family number
POLYMER MATERIAL CONTAINING CHEMICALLY MODIFIED POLYSACCHARIDE	ZUJUR DENISE [JP] KHAIRULINA KATERYNA [JP]	SMART TISSUES K K [JP]	WO2024214274A1	2023-04-14	A61K47/36 A61K9/48 C08B31/04 C08B37/00 C08B37/02 C08B37/08 C08F299/00 C08L5/00 C12M3/00	A61K47/36 (EP) A61K9/48 (EP) C08B31/04 (EP) C08B37/00 (EP) C08B37/0021 (EP) C08B37/003 (EP) C08F299/00 (EP) C08L5/00 (EP) C12M3/00 (EP)	2024-10-17	2024-10-17	093059014

7. CELLULAR SUPPORT FOR CULTURING METHODS

Title	Inventors	Applicants	Publication number	Earliest priority	IPC	CPC	Publication date	Earliest publication	Family number
CELLULAR SUPPORT FOR CULTURING METHODS	BASABE DESMONTS LOURDES [ES] BENITO LÓPEZ FERNANDO [ES] AZUAJE HUALDE ENRIQUE [ES] MARTÍNEZ DE PANCORBO GÓMEZ MARÍA DE LOS ÁNGELES [ES] ÁLVAREZ BRAÑA YARA [ES] ALONSO CABRERA JUNCAL [ES] LARTITEGUI MENESES NAIARA [ES] SAEZ CASTAÑO JANIRE [ES]	UNIV DEL PAIS VASCO/EUSKAL HERRIKO UNIBERTSITATEA [ES]	EP4435088A1	2023-03-22	C12M1/00 C12M1/12 C12M3/06	C12M23/16 (EP) C12M23/20 (EP) C12M25/06 (EP) C12M25/14 (EP) C12M25/16 (EP)	2024-09-25	2024-09-25	085726647

8. BIOPRINTING METHODS AND SYSTEMS

Title	Inventors	Applicants	Publication number	Earliest priority	IPC	CPC	Publication date	Earliest publication	Family number
BIOPRINTING	BEYER SIMON [CA]	ASPECT			B29C64/386	B29C64/106 (EP)			

METHODS AND SYSTEMS	WALUS KONRAD [CA] JAFARI MOHAMMAD HOSSEIN [CA]	BIOSYSTEMS LTD [CA]	WO2024178514A1	2023-03-01	B33Y10/00 B33Y30/00 B33Y50/00 C12M1/34 C12M1/36 C12M3/00 G06V10/82 G06V20/69 C12N5/00	B29C64/209 (EP) B29C64/393 (EP) B33Y10/00 (EP) B33Y30/00 (EP) B33Y50/02 (EP) C12M1/34 (EP) C12M1/36 (EP) C12M3/00 (EP) C12M33/00 (EP) G06V10/26 (EP) G06V10/469 (EP) G06V10/82 (EP) G06V20/69 (EP)	2024-09-06	2024-09-06	092589048

9. Granular gel with uniformly distributed pores as well as preparation method and application of granular gel

Title	Inventors	Applicants	Publication number	Earliest priority	IPC	CPC	Publication date	Earliest publication	Family number
Granular gel with uniformly distributed pores as well as preparation method and application of granular gel	YU ZIYI LIU YUE ZHANG JING CHEN CHENG	NANJING UNIVERSITY OF TECHNOLOGY	CN118440361A	2024-05-07	C08B37/08 C08F299/02 C08J3/24 C08L5/08 C08L55/00	C08B37/0072 (CN) C08F299/026 (CN) C08J3/246 (CN) C08J2305/08 (CN) C08J2405/08 (CN) C08J2455/00 (CN)	2024-08-06	2024-08-06	092332902

10. Bioink composition for preparing immunopolarized exosome laden 3D bioprinted hydrogel and use of the same

Title	Inventors	Applicants	Publication number	Earliest priority	IPC	CPC	Publication date	Earliest publication	Family number
Bioink composition for preparing immunopolarized exosome laden 3D bioprinted hydrogel and use of the same	LIM KI TAEK DEB DUTTA SAYAN	KNU INDUSTRY COOPERATION FOUND [KR]	KR20240114492A	2023-01-17	A61L27/24 A61L27/36 C09D11/04 C12N5/0786	A61L27/24 (KR) A61L27/3633 (KR) A61L27/52 (KR) C09D11/04 (KR) C12N5/0645 (KR)	2024-07-24	2024-07-24	092171180

11. Preparation method of self-healing gelatin hydrogel and active hydrogel thereof

Title	Inventors	Applicants	Publication number	Earliest priority	IPC	CPC	Publication date	Earliest publication	Family number
Preparation method of self-healing gelatin hydrogel and active hydrogel thereof	WANG XIAOHONG	UNIV CHINA MEDICAL	CN118290947A	2024-05-11	C08J3/075 C08J3/24 C08L5/02 C08L89/00	C08J3/075 (CN) C08J3/24 (CN) C08J2389/00 (CN) C08J2405/02 (CN)	2024-07-05	2024-07-05	091677975

12. SYSTEMS AND METHODS FOR SEQUENCING NUCLEIC ACIDS FROM SINGLE EXTRACELLULAR VESICLES

Title	Inventors	Applicants	Publication number	Earliest priority	IPC	CPC	Publication date	Earliest publication	Family number
SYSTEMS AND METHODS FOR SEQUENCING NUCLEIC ACIDS FROM SINGLE EXTRACELLULAR VESICLES	SCHMITTGEN THOMAS D [US] ANGELINI THOMAS ETTOR [US] BROCK ANDREW [US] JIANG JINMAI [US] DURAIVEL SENTHILKUMAR [US]	UNIV FLORIDA [US]	WO2024118927A1	2022-12-02	B01D21/26 B05D1/04 B05D1/26 B05D7/02 C12N5/077 C12N5/09 C12N9/12 C12Q1/686	C12N5/0075 (EP) C12Q1/6844 (EP) C12Q1/6869 (EP) C12N2513/00 (EP) C12Q1/6844, C12Q2563/159, INV (EP) C12Q1/6869, C12Q2563/159, INV (EP)	2024-06-06	2024-06-06	091325049

13. Double-layer hydrogel bioactive scaffold as well as preparation method and application thereof

Title	Inventors	Applicants	Publication number	Earliest priority	IPC	CPC	Publication date	Earliest publication	Family number
Double-layer hydrogel bioactive scaffold as well as preparation method and application thereof	YANG LEI HUANG JIANGHONG LIANG YUJIE	SHENZHEN SECOND PEOPLES HOSPITAL SHENZHEN INST OF	CN118045235A	2024-02-04	A61L27/20 A61L27/22 A61L27/40 A61L27/52	A61L27/20 (CN) A61L27/222 (CN) A61L27/52 (CN) A61L27/56 (CN)	2024-05-17	2024-05-17	091053117
		TRANSLATIONAL MEDICINE			A61L27/56	A61L2430/06 (CN) A61L2430/24 (CN) A61L27/20, C08L5/04, INV (CN)			

14. Bio-pen structure capable of controlling bio-ink or hydrogel particle size and bio-printing method using the same

Title	Inventors	Applicants	Publication number	Earliest priority	IPC	CPC	Publication date	Earliest publication	Family number
Bio-pen structure capable of controlling bio-ink or hydrogel particle size and bio-printing method using the same	INSUP NOH AMITAVA BHATTACHARYYA	MATRIXCELL BIO CO LTD [KR] FOUND RES & BUSINESS SEOUL NAT UNIV SCI & TECH [KR]	KR20240024730A	2022-08-16	B29C48/285 B29C48/30 B29C48/40 B29C48/68 B29C64/209 B29C64/321 B29C64/393 B33Y30/00 B33Y40/00 B33Y50/02 C12M1/00	B29C48/285 (KR) B29C48/30 (KR) B29C48/40 (KR) B29C48/682 (KR) B29C64/209 (KR) B29C64/232 (KR) B29C64/236 (KR) B29C64/241 (KR) B29C64/245 (KR) B29C64/25 (KR) B29C64/321 (KR) B29C64/393 (KR) B33Y30/00 (KR) B33Y40/00 (KR) B33Y50/02 (KR) B33Y70/00 (KR) C12M29/06 (KR)	2024-02-26	2024-02-26	090058180

15. Umbilical cord mesenchymal stem cell exosome scaffold as well as preparation method and application thereof

Title	Inventors	Applicants	Publication number	Earliest priority	IPC	CPC	Publication date	Earliest publication	Family number
Umbilical cord mesenchymal stem cell exosome scaffold as well as preparation method and application thereof	HUANG JIANGHONG YANG LEI LIANG YUJIE	SHENZHEN SECOND PEOPLES HOSPITAL SHENZHEN INST OF TRANSLATIONAL MEDICINE	CN117448265A	2023-08-31	A61L31/00 A61L31/04 A61L31/14 A61L31/16 C12N5/0775	A61L31/005 (CN) A61L31/042 (CN) A61L31/045 (CN) A61L31/145 (CN) A61L31/146 (CN) A61L31/16 (CN) C12N5/0668 (CN) A61L2300/412 (CN) A61L2300/602 (CN) A61L2430/40 (CN) C12N2509/00 (CN) C12N2509/10 (CN) C12N2533/54 (CN) C12N2533/74 (CN) C12N2535/00 (CN)	2024-01-26	2024-01-26	089591721

						A61L31/042, C08L5/04, INV (CN)			

16. PRINTING OF CELL AGGREGATES

Title	Inventors	Applicants	Publication number	Earliest priority	IPC	CPC	Publication date	Earliest publication	Family number
PRINTING OF CELL AGGREGATES	LUYTEN FRANK [BE] PAPANTONIOU IOANNIS [BE] IAZZOLINO ANTONIO [FR] VIELLEROBE BERTRAND [FR] GUILLEMOT FABIEN [FR]	UNIV LEUVEN KATH [BE] POIETIS [FR]	WO2023247801A1	2022-06-24	C12M1/26 C12M3/00	C12M21/08 (EP) C12M33/00 (EP)	2023-12-28	2023-12-28	082308492

17. DEVICES AND METHODS OF PRODUCING TUBULAR SYSTEMS FOR CELL CULTURE

Title	Inventors	Applicants	Publication number	Earliest priority	IPC	CPC	Publication date	Earliest publication	Family number
DEVICES AND METHODS OF PRODUCING TUBULAR SYSTEMS FOR CELL CULTURE	XIE YUBING [US] JORGENSEN MATTHEW [US] KOLLAMPALLY SUJITH [US]	RESEARCH FOUNDATION FOR THE STATE UNIV OF NEW YORK [US]	US2023374446A1	2020-01-20	C12M1/00 C12N5/00	C12M25/14 (EP) C12M29/00 (US) C12N5/0062 (EP) C12N5/0068 (US) C12N5/0621 (EP) C12N2501/115 (EP) C12N2513/00 (US) C12N2533/74 (EP) C12N2537/10 (EP,US)	2023-11-23	2023-11-23	088792246

18. Preparation method of 3D printing double-layer nano composite hydrogel scaffold for comprehensive repair of cartilage and bone

Title	Inventors	Applicants	Publication number	Earliest priority	IPC	CPC	Publication date	Earliest publication	Family number
Preparation method of 3D printing double-layer nano composite hydrogel scaffold for comprehensive repair of cartilage and bone	FENG LONGBAO GUO RUI	GUANGZHOU BEOGENE BIOTECHNOLOGY CO LTD	CN117018278A	2023-09-04	A61L27/12 A61L27/22 A61L27/52 A61L27/54 B33Y10/00 B33Y70/10	A61L27/12 (CN) A61L27/222 (CN) A61L27/52 (CN) A61L27/54 (CN) B33Y10/00 (CN) B33Y70/10 (CN) A61L2300/412 (CN) A61L2300/608 (CN) A61L2430/02 (CN) A61L2430/06 (CN) A61L27/222, C08L89/00, INV (CN)	2023-11-10	2023-11-10	088631802

19. ENZYME-FREE PROCESSES TO PRODUCE HYDROGELS

Title	Inventors	Applicants	Publication number	Earliest priority	IPC	CPC	Publication date	Earliest publication	Family number
ENZYME-FREE PROCESSES TO PRODUCE HYDROGELS	LIGUORI GABRIEL ROMERO [BR] LIGUORI TÁCIA TAVARES DE AQUINAS [BR] DE SOUZA FERNANDA CARLA BOMBALDI [BR] DE SOUZA RENATA	TISSUELABS PESQUISA E DESENVOLVIMENTO LTDA [BR]	EP4506020A2	2022-04-07	A61K35/12 A61K47/06 A61K47/30 A61L27/14 A61L27/36		2025-02-12	2023-10-12	088244222

	FRANCIELLE BOMBALDI [BR]								

20. ADAPTIVE PATCHES FOR DYNAMIC ORGANS

Title	Inventors	Applicants	Publication number	Earliest priority	IPC	CPC	Publication date	Earliest publication	Family number
ADAPTIVE PATCHES FOR DYNAMIC ORGANS	NGUYEN JULIANE [US] CHANSORIA PARTH [US] EGAN TOM [US]	UNIV NORTH CAROLINA CHAPEL HILL [US]	WO2023196236A2 WO2023196236A3	2022-04-04	A61F13/00 A61K9/70 A61F13/02 A61L27/40 B29C44/34 B29C70/68	A61F13/00063 (EP) A61F13/01017 (EP) A61F13/01021 (EP) A61K45/00 (EP) A61K47/32 (EP) A61K47/42 (EP) A61K9/0014 (EP) A61K9/06 (EP) A61K9/7007 (EP)	2023-10-12 2023-11-23	2023-10-12	088243401

21. COLLAGEN-POLYMER SCAFFOLD DELIVERY SYSTEM FOR PERIODONTAL REPAIR AND REGENERATION

Title	Inventors	Applicants	Publication number	Earliest priority	IPC	CPC	Publication date	Earliest publication	Family number
COLLAGEN-POLYMER SCAFFOLD DELIVERY SYSTEM FOR PERIODONTAL REPAIR AND REGENERATION	NJIKANG GABRIEL N [US] LE ALAN NGOC [US] LUBIT BEVERLY W [US]	RVO 2 0 INC D/B/A OPTICS MEDICAL [US]	US2023293772A1	2022-03-21	A61L27/26 A61L27/38 A61L27/52 A61L27/56 A61L27/58	A61L27/26 (EP,US) A61L27/3834 (EP,US) A61L27/3865 (EP,US) A61L27/52 (EP,US) A61L27/54 (EP) A61L27/56 (EP,US) A61L27/58 (EP,US) A61L2300/414 (EP,US) A61L2400/06 (EP,US) A61L27/26, C08L89/06, INV (EP)	2023-09-21	2023-09-21	088066190

22. SYSTEMS, COMPOSITIONS AND METHODS FOR LOW TEMPERATURE PRESERVATION OF CELLS, BIOINKS, HYDROGELS, AND TISSUE ENGINEERED MEDICINAL PRODUCTS (TEMPS)

Title	Inventors	Applicants	Publication number	Earliest priority	IPC	CPC	Publication date	Earliest publication	Family number
SYSTEMS, COMPOSITIONS AND METHODS FOR LOW TEMPERATURE PRESERVATION OF CELLS, BIOINKS, HYDROGELS, AND TISSUE ENGINEERED MEDICINAL PRODUCTS (TEMPS)	PRABHATHACHANDRAN ANUPAMA [US] BHUIYAN DIDARUL B [US] LI RUI [US] LYNESS ALEXANDER M [US] MATAKAS WILLIAM B [US]	WEST PHARMACEUTICAL SERVICES INC [US]	WO2023168387A1	2022-03-04	A61L27/16 A61L27/18 A61L27/20 A61L27/22 A61L27/24 A61L27/38 A61L27/52 A61L27/54 B33Y10/00 B33Y70/10	A61L27/16 (EP) A61L27/18 (EP) A61L27/20 (EP) A61L27/222 (EP) A61L27/225 (EP) A61L27/24 (EP) A61L27/3834 (EP) A61L27/52 (EP) A61L27/54 (EP) B33Y10/00 (EP) B33Y70/10 (EP) A61L2300/254 (EP) A61L2300/414	2023-09-07	2023-09-07	085800607

						(EP) A61L2300/802 (EP) A61L27/16, C08L33/10, INV (EP) A61L27/16, C08L39/06, INV (EP) A61L27/18, C08L67/04, INV (EP) A61L27/18, C08L71/02, INV (EP) A61L27/20, C08L5/02, INV (EP) A61L27/20, C08L5/04, INV (EP) A61L27/20, C08L5/08, INV (EP) A61L27/20, C08L5/12, INV (EP)			

23. 3D printing biological material suitable for scar-free repair of diabetic wound and preparation method of 3D printing biological material

Title	Inventors	Applicants	Publication number	Earliest priority	IPC	CPC	Publication date	Earliest publication	Family number
3D printing biological material suitable for scar-free repair of diabetic wound and preparation method of 3D printing biological material	HU YANKE XIONG YAHUI ZHOU FEI CHEN LEI QI SHAOHAI	THE FIRST AFFILIATED HOSPITAL OF SUN YAT SEN UNIV	CN116672504A	2023-07-19	A61L27/04 A61L27/36 A61L27/38 A61L27/50 A61L27/52 A61L27/54 A61L27/58 A61L27/60 B33Y10/00 B33Y70/10 B33Y80/00	A61L27/047 (CN) A61L27/3633 (CN) A61L27/3834 (CN) A61L27/50 (CN) A61L27/52 (CN) A61L27/54 (CN) A61L27/58 (CN) A61L27/60 (CN) B33Y10/00 (CN) B33Y70/10 (CN) B33Y80/00 (CN) A61L2300/102 (CN) A61L2300/216 (CN) A61L2300/40 (CN) A61L2300/41 (CN) A61L2300/412 (CN) A61L2300/602 (CN)	2023-09-01	2023-09-01	087791152

24. BIOPRINTER AND RELATED SYSTEMS AND COMPONENTS

Title	Inventors	Applicants	Publication number	Earliest priority	IPC	CPC	Publication date	Earliest publication	Family number
BIOPRINTER AND RELATED SYSTEMS AND COMPONENTS	HILLMAN AVRIEL [US] LIEBSCHENER MICHAEL [US] ALEXANDER PETER [US]	BIOHIP CORP [US]	WO2024167956A1	2022-02-04	A61F2/02 B29C64/10 B33Y10/00 B33Y30/00	A61F2/02 (US) B29C64/106 (EP) B29C64/112 (EP,US) B29C64/209 (EP) B29C64/227 (EP)	2024-08-15	2023-08-10	087521437

						B29C64/241 (EP) B33Y10/00 (EP) B33Y30/00 (EP,US) B33Y80/00 (EP) A61F2/02 (EP) A61F2240/00 (US) A61F2240/002 (EP)			

25. BIOFABRICATION OF A TRI-LAYERED 3D-BIOPRINTED CSC-BASED MALIGNANT MELANOMA MODEL

Title	Inventors	Applicants	Publication number	Earliest priority	IPC	CPC	Publication date	Earliest publication	Family number
BIOFABRICATION OF A TRI-LAYERED 3D-BIOPRINTED CSC-BASED MALIGNANT MELANOMA MODEL	MARCHAL CORRALES JUAN ANTONIO [ES] JIMÉNEZ GONZÁLEZ GEMA [ES] LÓPEZ DE ANDRÉS JULIA [ES]	UNIV GRANADA [ES]	EP4223869A1	2022-02-05	A61L27/38 A61L27/60 B33Y80/00 C12N5/071	A61L27/26 (EP) A61L27/3808 (EP) A61L27/3813 (EP) A61L27/3834 (EP) A61L27/52 (EP) A61L27/60 (EP) B33Y10/00 (EP) B33Y70/00 (EP) B33Y80/00 (EP) C12N5/0698 (EP) C12N2502/091 (EP) C12N2502/092 (EP) C12N2502/094 (EP) C12N2502/1305 (EP) C12N2502/1323 (EP) C12N2513/00 (EP) C12N2533/54 (EP) C12N2533/70 (EP) A61L27/26, C08L5/12, INV (EP) A61L27/26, C08L89/06, INV (EP)	2023-08-09	2023-08-09	080683239

26. TINTAS DE REDE DUPLA DE GEL COLOIDAL PARA IMPRESSÃO 3D, COMPONENTES, MÉTODOS DE PRODUÇÃO E USOS DOS MESMOS

Title	Inventors	Applicants	Publication number	Earliest priority	IPC	CPC	Publication date	Earliest publication	Family number
TINTAS DE REDE DUPLA DE GEL COLOIDAL PARA IMPRESSÃO 3D, COMPONENTES, MÉTODOS DE PRODUÇÃO E USOS DOS MESMOS	VÍTOR MANUEL ABREU GASPAR [PT] JOÃO FILIPE COLARDELLE DA LUZ MANO [PT] PEDRO OLIVEIRA LAVRADOR [PT] LEANDRO DOS SANTOS GONÇALVES [PT]	UNIV AVEIRO [PT]	PT117637A	2021-12-15	A61K47/00 A61L27/00 B33Y70/00 B33Y80/00	A61K47/00 (PT) A61L27/00 (PT) B33Y70/00 (PT) B33Y80/00 (PT)	2023-07-24	2023-07-24	087428248

27. INTERLOCKING POROUS HYDROGEL BLOCKS

Title	Inventors	Applicants	Publication number	Earliest priority	IPC	CPC	Publication date	Earliest publication	Family number
INTERLOCKING POROUS HYDROGEL BLOCKS	MELLOTT ADAM<br>HODGE JACOB G<br>DECKER HEATHER E	RONAWK INC [US]	AU2023205010A1	2022-01-04	C12N5/00<br>C08B37/08<br>C12M1/32	C12M25/14 (EP,IL)<br>C12N5/0629 (EP,IL)<br>C12N5/0667 (EP,IL)<br>C12N2513/00 (EP,IL)<br>C12N2533/40 (EP,IL)<br>C12N2533/52 (EP,IL)<br>C12N2535/10 (EP,IL)<br>C12N2537/10 (EP,IL)	2024-07-11	2023-07-13	087074174

28. CELL CULTIVATION METHODOLOGY

Title	Inventors	Applicants	Publication number	Earliest priority	IPC	CPC	Publication date	Earliest publication	Family number
CELL CULTIVATION METHODOLOGY	MELLOTT ADAM J<br>HODGE JACOB G<br>DECKER HEATHER E	RONAWK INC [US]	AU2023205537A1	2022-01-04	C12M3/00<br>C12M1/18<br>C12N5/071	C12M25/14 (EP,IL)<br>C12N5/0068 (EP,IL)<br>C12N5/0629 (EP,IL)<br>C12N5/0667 (EP,IL)<br>C12N2500/50 (EP,IL)<br>C12N2513/00 (EP,IL)<br>C12N2539/00 (EP,IL)	2024-07-11	2023-07-13	087074180

29. APPLICATIONS OF BIOLOGICAL BLOCK PLATFORM

Title	Inventors	Applicants	Publication number	Earliest priority	IPC	CPC	Publication date	Earliest publication	Family number
APPLICATIONS OF BIOLOGICAL BLOCK PLATFORM	MELLOTT ADAM J<br>HODGE JACOB G<br>DECKER HEATHER E	RONAWK INC [US]	AU2023205180A1	2022-01-04	A61L27/52<br>A61L27/46<br>A61L27/54<br>A61L27/60	A61L27/38 (EP,IL)<br>A61L27/52 (EP,IL)<br>A61L27/56 (EP,IL)	2024-07-11	2023-07-13	087074151

30. Bioreactors and related apparatuses

Title	Inventors	Applicants	Publication number	Earliest priority	IPC	CPC	Publication date	Earliest publication	Family number
Bioreactors and related apparatuses	INSUP NOH AMITAVA BHATTACHARYYA	MATRIXCELL BIO CO LTD [KR] FOUND RES & BUSINESS SEOUL NAT UNIV SCI & TECH [KR]	KR20230104064A	2021-12-30	C12M1/12<br>C12M1/32<br>C12M1/42<br>C12M3/00	C12M23/12 (KR)<br>C12M23/42 (KR)<br>C12M35/02 (KR)<br>C12M35/04 (KR)<br>C12M37/00 (KR)	2023-07-07	2023-07-07	087154376

31. BIOREACTOR AND ASSOCIATED DEVICE

Title	Inventors	Applicants	Publication number	Earliest priority	IPC	CPC	Publication date	Earliest publication	Family number
BIOREACTOR AND ASSOCIATED DEVICE	NOH INSUP [KR]<br>BHATTACHARYYA AMITAVA [KR]	MATRIXCELL BIO CO LTD [KR] FOUND RES & BUSINESS SEOUL NAT UNIV SCI & TECH [KR]	WO2023128714A1	2021-12-30	C12M1/00<br>C12M1/32<br>C12M1/42<br>C12M3/00	C12M1/00 (EP)<br>C12M1/32 (EP)<br>C12M1/42 (EP)<br>C12M3/00 (EP)	2023-07-06	2023-07-06	086999801

32. Method for preparing exosome through low-oxygen culture of stem cells based on biological 3D printing

Title	Inventors	Applicants	Publication number	Earliest priority	IPC	CPC	Publication date	Earliest publication	Family number
Method for preparing exosome through low-oxygen culture of stem cells based on biological 3D printing	TAN JICHUN YANG ZU ZHANG SIWEN LIN CHAO ZENG YANHUA LIU NA GAO SHAN	SUZHOU YUANSHENG CELL BIOTECHNOLOGY CO LTD	CN116333978A	2023-03-29	C12N5/0775	C12N5/0665 (CN) C12N2500/02 (CN) C12N2500/20 (CN) C12N2500/30 (CN) C12N2500/32 (CN) C12N2500/46 (CN) C12N2501/105 (CN) C12N2501/11 (CN) C12N2501/115 (CN) C12N2513/00 (CN) C12N2533/30 (CN) C12N2533/54 (CN) C12N2533/56 (CN) C12N2533/70 (CN) C12N2533/72 (CN) C12N2533/74 (CN) Y02P10/25 (EP)	2023-06-27	2023-06-27	086883680

33. BIO-PEN STRUCTURE FOR IMPROVING MIXING HOMOGENEITY AND BIO-PRINTING METHOD USING THE SAME

Title	Inventors	Applicants	Publication number	Earliest priority	IPC	CPC	Publication date	Earliest publication	Family number
BIO-PEN STRUCTURE FOR IMPROVING MIXING HOMOGENEITY AND BIO-PRINTING METHOD USING THE SAME	NOH INSUP [KR] BHATTACHARYYA AMITAVA [KR]	MARRIXCELL BIO CO LTD [KR] FOUND RES & BUSINESS SEOUL NAT UNIV SCI & TECH [KR]	US2025042081A1	2021-12-06	B29C48/02 B29C48/25 B29C48/40 B29C48/435 B29C48/535 B29C48/68 B29C64/118 B29C64/209 B29C64/218 B29C64/268 B29C64/321 B33Y10/00 B33Y30/00 B33Y80/00 B29K105/00 B29L31/00	B29C48/02 (EP,US) B29C48/2526 (EP,US) B29C48/285 (EP) B29C48/30 (EP) B29C48/40 (EP) B29C48/402 (EP,US) B29C48/435 (EP,US) B29C48/535 (EP,US) B29C48/68 (EP) B29C48/682 (EP,US) B29C64/106 (EP) B29C64/118 (US) B29C64/209 (EP,US) B29C64/218 (US)	2025-02-06	2023-06-15	086730877

						B29C64/268 (US) B29C64/321 (EP,US) B33Y10/00 (EP,US) B33Y30/00 (EP,US) B33Y40/00 (EP) B33Y70/00 (EP) C12M1/00 (EP) B29K2105/0061 (EP,US) B29L2031/7532 (EP,US) B33Y80/00 (EP,US)			

34. COMPOSITIONS AND METHODS FOR PRODUCTION AND USE OF A SCALABLE HUMAN CELL-DERIVED EXTRACELLULAR MATRIX

Title	Inventors	Applicants	Publication number	Earliest priority	IPC	CPC	Publication date	Earliest publication	Family number
COMPOSITIONS AND METHODS FOR PRODUCTION AND USE OF A SCALABLE HUMAN CELL-DERIVED EXTRACELLULAR MATRIX	SKARDAL ALEKSANDER [US]	OHIO STATE INNOVATION FOUNDATION [US]	US2025034530A1	2021-12-06	C12N5/077 C12N5/09	C12N5/0062 (EP) C12N5/0068 (EP) C12N5/0656 (US) C12N5/0693 (US) C12N2502/1323 (EP) C12N2502/30 (EP) C12N2531/00 (US) C12N2533/30 (EP) C12N2533/54 (US) C12N2533/80 (EP) C12N2533/90 (EP,US) C12N2537/10 (EP,US) C12N5/0075 (EP) C12N5/0656 (EP)	2025-01-30	2023-06-15	086731261

35. MICROPHYSIOLOGICAL SYSTEM AND USES THEREOF

Title	Inventors	Applicants	Publication number	Earliest priority	IPC	CPC	Publication date	Earliest publication	Family number
MICROPHYSIOLOGICAL SYSTEM AND USES THEREOF	HAMEL DIMITRI [FR] FERRAND AUDREY [FR] FONCY JULIE [FR] MALAQUIN LAURENT [FR]	INST NAT SANTE RECH MED [FR] CENTRE NAT RECH SCIENT [FR] UNIV TOULOUSE 3 PAUL SABATIER [FR] ECOLE NAT VETERINAIRE DE TOULOUSE [FR] INSTITUT NATIONAL DE RECH POUR L'AGRICULTURE L'ALIMENTATION ET	WO2023104915A1	2021-12-08	C12M1/00 C12M1/12 C12M3/00 C12M3/06	C12M21/08 (EP) C12M23/16 (EP) C12M23/34 (EP) C12M25/02 (EP)	2023-06-15	2023-06-15	078957952

		L'ENVIRONNEMENT [FR]							

36. BIO-PEN STRUCTURE FOR IMPROVING MIXING HOMOGENEITY, AND BIO-PRINTING MEHOD USING SAME

Title	Inventors	Applicants	Publication number	Earliest priority	IPC	CPC	Publication date	Earliest publication	Family number
BIO-PEN STRUCTURE FOR IMPROVING MIXING HOMOGENEITY, AND BIO-PRINTING MEHOD USING SAME	NOH INSUP [KR] BHATTACHARYYA AMITAVA [KR]	MATRIXCELL BIO CO LTD [KR] FOUND RES & BUSINESS SEOUL NAT UNIV SCI & TECH [KR]	EP4446093A1	2021-12-06	B29C48/285 B29C48/30 B29C48/40 B29C48/68 B29C64/209 B29C64/321 B33Y30/00 B33Y40/00 B33Y70/00 C12M1/00	B01F23/812 (EP) B01F27/1143 (EP) B01F27/722 (EP) B01F27/922 (EP) B01F27/923 (EP) B29C48/02 (EP) B29C48/285 (EP,KR) B29C48/297 (EP) B29C48/30 (KR) B29C48/40 (KR) B29C48/42 (EP) B29C48/682 (KR) B29C64/106 (EP) B29C64/209 (EP,KR) B29C64/321 (EP,KR) B33Y10/00 (EP) B33Y30/00 (EP,KR) B33Y40/00 (EP,KR) B33Y70/00 (EP,KR) C12M29/06 (KR) B01F2215/0422 (EP) B01F2215/0431 (EP) C12M33/00 (EP)	2024-10-16	2023-06-13	086762889

37. Bio-ink composition containing giant salamander skin secretions and application thereof

Title	Inventors	Applicants	Publication number	Earliest priority	IPC	CPC	Publication date	Earliest publication	Family number
Bio-ink composition containing giant salamander skin secretions and application thereof	ZHANG XIMU	STOMATOLOGICAL HOSPITAL OF CHONGQING MEDICAL UNIV	CN115998954A CN115998954B	2021-10-22	A61L27/16 A61L27/18 A61L27/20 A61L27/22 A61L27/24 A61L27/36 A61L27/38 A61L27/54 A61L27/60 B33Y70/10 C08B37/08 C08H1/00		2023-04-25 2023-10-20	2023-04-25	086028541

38. Gelatin/sodium alginate hydrogel-based 3D printing bio-ink and application thereof

Title	Inventors	Applicants	Publication number	Earliest priority	IPC	CPC	Publication date	Earliest publication	Family number
Gelatin/sodium alginate hydrogel-based 3D printing bio-ink and application thereof	MAO HONGLI GU ZHONGWEI HAO LILI ZHAO SHIJIA	NANJING UNIVERSITY OF TECHNOLOGY	CN115887772A CN115887772B	2022-11-17	A61L27/20 A61L27/22 A61L27/38 A61L27/52	Y02P20/54 (EP)	2023-04-04 2024-12-03	2023-04-04	086492771

					A61L27/60 B33Y10/00 B33Y70/10				

39. MICROFLUIDIC-BASED FIBER FORMATION METHODS AND SYSTEMS

Title	Inventors	Applicants	Publication number	Earliest priority	IPC	CPC	Publication date	Earliest publication	Family number
MICROFLUIDIC-BASED FIBER FORMATION METHODS AND SYSTEMS	BEYER SIMON [CA] WADSWORTH SAMUEL [CA] HE JACKSON [CA] XU ZHENSONG [CA] KHAN USAMA [CA] WALUS KONRAD [CA] SEPEHRI ANOUSH [CA] RODRIGUEZ MARITA [CA]	ASPECT BIOSYSTEMS LTD [CA]	CA3230168A1	2021-08-27	B29C64/393	B29C64/106 (EP,IL,KR) B29C64/209 (EP,IL,KR) B29C64/343 (EP,IL,KR) B29C64/393 (EP,IL,KR) B33Y10/00 (EP,IL,KR) B33Y30/00 (EP,IL,KR) B33Y40/00 (KR) B33Y50/02 (EP,IL,KR) B33Y70/10 (EP,IL,KR) G01F1/704 (EP,IL,KR) G06N3/044 (IL,KR) G06N3/0464 (IL,KR) G06N3/084 (IL,KR) G06T17/00 (KR) H04N7/18 (KR) B29C2037/906 (KR) G06N3/044 (EP) G06N3/0464 (EP) G06N3/084 (EP)	2023-03-02	2023-03-02	085321424

40. METHODS FOR PRODUCTION OF ENGINEERED CELLS

Title	Inventors	Applicants	Publication number	Earliest priority	IPC	CPC	Publication date	Earliest publication	Family number
METHODS FOR PRODUCTION OF ENGINEERED CELLS	CHASTAGNIER LAURA [FR] PETIOT EMMA [FR]	SARTORIUS STEDIM FMT S A S [FR] UNIV CLAUDE BERNARD LYON | [FR] INSTITUT NAT DES SCIENCES APPLIQUEES DE LYON [FR] ECOLE SUPERIEURE DE CHIMIE PHYSIQUE ELECTRONIQUE DE LYON [FR] CENTRE NATIONAL DE LA RECHERCHE SCIENT [FR]	US2025051719A1	2021-08-25	B33Y10/00 B33Y70/00 C12N5/00 C12P1/00	B33Y10/00 (EP,US) B33Y70/00 (EP,US) B33Y80/00 (EP) C12M25/14 (EP) C12M33/00 (EP) C12N5/0062 (US) C12P1/00 (US) C12N2501/02 (US) C12N2513/00 (US)	2025-02-13	2023-03-01	077750187

41. METHODS FOR CULTURING MESENCHYMAL STEM CELLS, COMPOSITIONS AND IMPLEMENTATIONS THEREOF

Title	Inventors	Applicants	Publication number	Earliest priority	IPC	CPC	Publication date	Earliest publication	Family number
METHODS FOR CULTURING MESENCHYMAL STEM CELLS, COMPOSITIONS AND IMPLEMENTATIONS THEREOF	BHOWMICK TUHIN [US] CHANDRU ARUN [IN] THOMAS MIDHUN BEN [IN] SENGUPTA SUMAN [IN] MENON DEEPTHI [IN] KARUNAKARAN WENSON RAJAN DAVID [IN] SELVAM SHIVARAM [IN]	PANDORUM TECH PRIVATE LIMITED [IN]	US2024408143A1	2021-08-11	A61K35/28 A61P27/02 C12N5/0775	A61K35/28 (KR,US) A61K9/0048 (KR) A61P1/16 (KR) A61P27/02 (KR,US) C12N5/0663 (EP,KR,US) C12N2500/98 (US) C12N2501/60 (US) C12N2502/085 (EP) C12N2509/00 (KR)	2024-12-12	2023-02-16	083507459

42. PROCESS TO OBTAIN THREE-DIMENSIONAL BIODRESSING, THREE- DIMENSIONAL BIODRESSING OBTAINED AND ITS USE

Title	Inventors	Applicants	Publication number	Earliest priority	IPC	CPC	Publication date	Earliest publication	Family number
PROCESS TO OBTAIN THREE-DIMENSIONAL BIODRESSING, THREE-DIMENSIONAL BIODRESSING OBTAINED AND ITS USE	OLIVEIRA CAROLINA CALIARI [BR] MANFIOLLI ADRIANA OLIVEIRA [BR]	IN SITU TERAPIA CELULAR LTDA [BR]	CA3227390A1	2021-07-30	A61L15/28 A61L15/32 A61L15/44	A61L15/28 (EP) A61L15/32 (EP) A61L15/44 (EP) A61L26/0023 (EP) A61L26/0033 (EP) A61L26/0038 (EP) A61L26/0066 (EP) B33Y10/00 (EP) B33Y80/00 (EP) A61L2300/64 (EP) A61L15/28, C08L1/00, INV (EP) A61L15/28, C08L5/04, INV (EP) A61L15/28, C08L5/08, INV (EP) A61L15/32, C08L89/06, INV (EP) A61L26/0023, C08L1/00, INV (EP) A61L26/0023, C08L5/04, INV (EP) A61L26/0023, C08L5/08, INV (EP)	2023-02-02	2023-02-02	082942312

43. THREE-DIMENSIONAL TUMOR MODEL OF GLIOBLASTOMA AND BRAIN METASTASIS, METHODS OF MANUFACTURING SAME A ND USES THEREOF

Title	Inventors	Applicants	Publication number	Earliest priority	IPC	CPC	Publication date	Earliest publication	Family number
THREE-DIMENSIONAL	SATCHI-FAINARO RONIT	UNIV RAMOT [IL]			C12N5/071	B29C64/124 (EP)			

TUMOR MODEL OF GLIOBLASTOMA AND BRAIN METASTASIS, METHODS OF MANUFACTURING SAME AND USES THEREOF	[IL] NEUFELD LENA [IL]		US2024167001A1	2021-07-29		B33Y10/00 (EP) B33Y70/10 (EP) B33Y80/00 (EP) C12M25/14 (EP) C12M29/10 (EP) C12N5/0693 (EP) C12N5/0697 (EP,US) G01N33/5011 (EP) G01N33/5058 (EP) G01N33/5082 (EP) G09B23/30 (EP) C12N2501/115 (EP) C12N2502/086 (EP) C12N2502/28 (EP) C12N2503/02 (EP,US) C12N2513/00 (EP,US) C12N2533/54 (EP) C12N2533/56 (EP) C12N2537/10 (EP) C12N5/0622 (EP) C12N5/069 (EP) G01N2800/52 (EP)	2024-05-23	2023-02-02	085086443

44. OSTEOSARCOMA ORGANOID MODEL, CONSTRUCTION METHOD, AND USE

Title	Inventors	Applicants	Publication number	Earliest priority	IPC	CPC	Publication date	Earliest publication	Family number
OSTEOSARCOMA ORGANOID MODEL, CONSTRUCTION METHOD, AND USE	LU ZUYAN [CN] DAI KERONG [CN]	SHANGHAI 9TH PEOPLES HOSPITAL SHANGHAI JIAOTONG UNIV SCHOOL MEDICINE [CN]	WO2024012073A1	2022-07-14	B01L3/00 C12M1/00 C12M3/00 C12N5/077 C12N5/0775 C12N5/09 C12Q1/02	B01L3/00 (EP) C12M1/00 (EP) C12M3/00 (EP) C12N5/06 (EP) C12Q1/02 (EP)	2024-01-18	2023-01-31	085023493

45. 3D-PRINTABLE SHEAR-THINNING POLYSACCHARIDE-BASED NANOCOMPOSITE HYDROGEL FOR BIOMIMETIC TISSUE ENGINEERING

Title	Inventors	Applicants	Publication number	Earliest priority	IPC	CPC	Publication date	Earliest publication	Family number
3D-PRINTABLE SHEAR-THINNING POLYSACCHARIDE-BASED NANOCOMPOSITE HYDROGEL FOR BIOMIMETIC TISSUE ENGINEERING	HE MEI [US]	UNIV FLORIDA [US]	US2024270942A1	2021-06-18	A61L26/00 B33Y70/00 C08L3/02	A61L26/0023 (US) A61L26/0033 (US) A61L26/0038 (US) A61L26/008 (US) B33Y70/00 (EP,US) B33Y80/00 (EP)	2024-08-15	2022-12-22	084527611

						C08L3/02 (EP,US) A61L2400/12 (US) B29C64/106 (EP) B33Y10/00 (EP) C08L3/02, C08L89/06, INV (EP)			

46. Preparation and application of 3D bionic implant of IPS gene engineering cell

Title	Inventors	Applicants	Publication number	Earliest priority	IPC	CPC	Publication date	Earliest publication	Family number
Preparation and application of 3D bionic implant of IPS gene engineering cell	LI SHIYU SUN HANXIAO DENG JIANSHAN	AIWEIYA GUANGZHOU PHARMACEUTICAL TECH CO LTD	CN115463266A	2021-06-12	A61L31/00 A61L31/06 A61L31/14 A61L31/16 C12N5/10	A61L31/005 (CN) A61L31/06 (CN) A61L31/14 (CN) A61L31/146 (CN) A61L31/16 (CN) C12N5/0696 (CN) C12N9/14 (CN) C12Y306/05 (CN) A61L2300/252 (CN) A61L2300/602 (CN) A61L2400/12 (CN) C12N2510/02 (CN) A61L31/06, C08L67/04, INV (CN)	2022-12-13	2022-12-13	084364929

47. SELECTIVELY PERMEABLE ANNULUS FIBERS AND METHODS OF USING SAME

Title	Inventors	Applicants	Publication number	Earliest priority	IPC	CPC	Publication date	Earliest publication	Family number
SELECTIVELY PERMEABLE ANNULUS FIBERS AND METHODS OF USING SAME	WADSWORTH SAM [CA] MOHAMED TAMER [CA] GETSIOS SPIRO [CA] RUSSO VALERIO [CA] PAN SHENG [CA] BEYER SIMON [CA] PIRET JAMES [CA] LOZANO RENE GONZALO PEDROZA [CA]	ASPECT BIOSYSTEMS LTD [CA]	CA3220261A1	2021-05-24	A61L27/24 A61L27/26 A61L27/50	A61L27/20 (EP,IL,KR) A61L27/54 (EP,IL,KR) B33Y80/00 (EP,IL,KR) C08L5/04 (IL,KR) A61L2300/62 (EP,IL,KR) A61L2300/64 (EP,IL,KR) A61L27/20, C08L5/04, INV (EP)	2022-12-01	2022-12-01	084228841

48. Bio-ink based on human-derived acellular matrix and preparation method and application thereof

Title	Inventors	Applicants	Publication number	Earliest priority	IPC	CPC	Publication date	Earliest publication	Family number
Bio-ink based on human-derived acellular matrix and preparation method and application thereof	CHENG LONG ZANG CHUANBAO LIU XIU XIN XIUXIU ZHAI WANG WANG YONGJIE	YINFENG CRYOGENIC MEDICAL TECH CO LTD YINFENG BIOLOGICAL GROUP LTD	CN115382020A CN115382020B	2022-09-21	A61L27/20 A61L27/22 A61L27/36 A61L27/50 B33Y10/00 B33Y70/10	A61L27/20 (CN) A61L27/222 (CN) A61L27/3604 (CN) A61L27/3687 (CN) A61L27/3691 (CN) A61L27/50 (CN) B33Y10/00 (CN) B33Y70/10	2022-11-25 2023-12-15	2022-11-25	084126553

						(CN) A61L27/20, C08L5/08, INV (CN) A61L27/20, C08L5/12, INV (CN) A61L27/222, C08L89/00, INV (CN)			

49. POLYSACCHARIDE HYDROGEL OPTICAL FIBERS AND THEIR FABRICATION AND USE

Title	Inventors	Applicants	Publication number	Earliest priority	IPC	CPC	Publication date	Earliest publication	Family number
POLYSACCHARIDE HYDROGEL OPTICAL FIBERS AND THEIR FABRICATION AND USE	DEMIRCI UTKAN [US] AHMED RAJIB [US] REIS RUI L [US] GUIMARAES CARLOS F [US]	UNIV LELAND STANFORD JUNIOR [US]	US2024184016A1	2021-04-23	A61K41/00 B82Y20/00 G01N21/64 G02B1/04 G02B6/02 G02B6/028	A61K41/0057 (US) G01N21/6428 (US) G02B1/046 (US) G02B1/048 (EP,US) G02B6/02033 (EP) G02B6/0229 (US) G02B6/0286 (US) B82Y20/00 (US) G01N2021/6439 (US) G02B1/048, C08L5/04, C08L101/14, INV (EP)	2024-06-06	2022-10-27	083722671

50. System and Method for Personalized Implantable Scaffolds for Wound Healing

Title	Inventors	Applicants	Publication number	Earliest priority	IPC	CPC	Publication date	Earliest publication	Family number
System and Method for Personalized Implantable Scaffolds for Wound Healing	PRABHAKAR ASHWIN [US]	PRABHAKAR ASHWIN [US]	US2022296424A1	2021-03-17	A61B5/00 A61F13/00 G06F30/23	A61B5/445 (EP,US) A61B5/4848 (EP) A61F13/00987 (EP,US) B29C64/386 (EP) B33Y50/00 (EP) B33Y80/00 (EP) G06F30/10 (EP) G06F30/23 (US) A61B5/01 (EP) A61B5/1079 (EP) A61B5/14539 (EP) A61F2013/00357 (EP,US) B33Y10/00 (EP) G06F2111/16 (EP) G06F2113/10 (EP)	2022-09-22	2022-09-22	083285735

51. SCAFFOLD PER LA RIGENERAZIONE TISSUTALE, IN PARTICOLARE PER LA RIGENERAZIONE OSSEA, E RELATIVO METODO DI FABBRICAZIONE

Title	Inventors	Applicants	Publication number	Earliest priority	IPC	CPC	Publication date	Earliest publication	Family number
SCAFFOLD PER LA RIGENERAZIONE TISSUTALE, IN PARTICOLARE PER LA	AURICCHIO FERDINANDO BARI ELIA CONTI MICHELE PERTEGHELLA SARA	PHARMAEXCEED S R L [IT] P4P S R L [IT]	IT202100005441A1	2021-03-09	A61L	A61L27/26 (EP,US) A61L27/3604 (US)	2022-09-09	2022-09-09	076034965

RIGENERAZIONE OSSEA, E RELATIVO METODO DI FABBRICAZIONE	SCOCOZZA FRANCA SORLINI MARZIO TORRE MARIA LUISA					A61L27/3687 (US) A61L27/3691 (US) A61L27/48 (EP) A61L27/52 (EP,US) A61L27/54 (EP,US) B33Y80/00 (EP) A61L2300/30 (EP,US) A61L2430/02 (EP,US) B33Y10/00 (EP) A61L27/26, C08L5/04, INV (EP) A61L27/26, C08L89/00, INV (EP) A61L27/48, C08L5/04, INV (EP) A61L27/48, C08L67/04, INV (EP) A61L27/48, C08L77/04, INV (EP)			

52. Method for preparing osteogenesis microenvironment from small molecular medicine and application of osteogenesis microenvironment

Title	Inventors	Applicants	Publication number	Earliest priority	IPC	CPC	Publication date	Earliest publication	Family number
Method for preparing osteogenesis microenvironment from small molecular medicine and application of osteogenesis microenvironment	TU XIAOLIN LIU YANGXI LI JUN WANG BO RUAN XIAOXIE CHEN JIE WANG XIAOFANG WANG PENGTAO	UNIV CHONGQING MEDICAL	CN114621915A	2022-02-25	A61L27/38 A61L27/50 A61L27/54 B33Y10/00 B33Y70/10 B33Y80/00 C12N5/077	A61L27/3821 (CN) A61L27/3847 (CN) A61L27/3895 (CN) A61L27/50 (CN) A61L27/54 (CN) B33Y10/00 (CN) B33Y70/10 (CN) B33Y80/00 (CN) C12N5/0654 (CN) A61L2300/412 (CN) A61L2430/02 (CN) C12N2501/415 (CN)	2022-06-14	2022-06-14	081899927

53. Mesenchymal stem cell scaffold as well as preparation method and application thereof

Title	Inventors	Applicants	Publication number	Earliest priority	IPC	CPC	Publication date	Earliest publication	Family number
Mesenchymal stem cell scaffold as well as preparation method and application thereof	HE LIHONG CHENG SHUTING ZHAO KAIFENG	UNIV SOOCHOW	CN119040257A	2022-03-28	A61L27/16 A61L27/18 A61L27/20 A61L27/22 A61L27/24 A61L27/38 A61L27/50	A61L27/16 (CN) A61L27/18 (CN) A61L27/20 (CN) A61L27/22 (CN) A61L27/222 (CN) A61L27/24 (CN) A61L27/3834	2024-11-29	2022-06-10	081867274

					C12N5/071<br>C12N5/0775	(CN) A61L27/50 (CN) C12N5/0663 (CN) C12N5/069 (CN) C12N2506/1353 (CN) C12N2513/00 (CN) C12N2533/54 (CN) C12N2533/90 (CN)			

54. Preparation method of 3D printing tracheal stent loaded with stem cell exosome

Title	Inventors	Applicants	Publication number	Earliest priority	IPC	CPC	Publication date	Earliest publication	Family number
Preparation method of 3D printing tracheal stent loaded with stem cell exosome	SHI HONGCAN SHEN ZHIMING LU DAN SUN FEI SHAN YIBO LU YI YUAN LEI ZHU JIANWEI	UNIV YANGZHOU	CN114533961A<br>CN114533961B	2022-02-28	A61L27/18<br>A61L27/22<br>A61L27/38<br>A61L27/50<br>A61L27/54<br>B33Y10/00<br>B33Y70/00<br>B33Y80/00<br>C12N5/0775	A61L27/18 (CN) A61L27/227 (CN) A61L27/3834 (CN) A61L27/3882 (CN) A61L27/50 (CN) A61L27/54 (CN) B33Y10/00 (CN) B33Y70/00 (CN) B33Y80/00 (CN) C12N5/0663 (CN) A61L2300/412 (CN) A61L2430/22 (CN) A61L2430/40 (CN) C12N2509/00 (CN) Y02P10/25 (EP) A61L27/18, C08L67/04, INV (CN) A61L27/227, C08L89/00, INV (CN)	2022-05-27<br>2023-01-31	2022-05-27	081679367

55. INTEGRATED 3D BIOPRINTING METHOD AND APPLICATION OF HARD MATERIALS AND CELLS FOR PREPARING BONE-REPAIR FUNCTIONAL MODULES AND BONE ORGANOIDS

Title	Inventors	Applicants	Publication number	Earliest priority	IPC	CPC	Publication date	Earliest publication	Family number
INTEGRATED 3D BIOPRINTING METHOD AND APPLICATION OF HARD MATERIALS AND CELLS FOR PREPARING BONE-REPAIR FUNCTIONAL MODULES AND BONE ORGANOIDS	TU XIAOLIN [CN] MA YUFEI [CN] LI JUN [CN] LI XIAN [CN] TANG ZHURONG [CN] XIE ZHENGSONG [CN] CHEN JIE [CN] WANG XIAOFANG [CN] LIU GUANGLIANG [CN] WANG PENGTAO [CN] WANG BO [CN] LUO YISHENG [CN] LIU YANGXI [CN] GONG WEIMIN [CN] LI MOLIN	UNIV CHONGQING MEDICAL [CN]	US2023104993A1	2021-09-30	A61L27/38<br>A61L27/46<br>A61L27/56<br>B33Y10/00<br>B33Y70/00<br>B33Y80/00<br>C12M3/00<br>C12N5/071<br>C12N5/077	A61L27/3821 (EP,US) A61L27/3834 (EP) A61L27/46 (EP,US) A61L27/56 (EP,US) B33Y10/00 (EP,US) B33Y30/00 (CN) B33Y70/00 (EP,US) B33Y80/00 (US)	2023-04-06	2022-03-22	080696061

	[CN]					C12M21/08 (EP,US) C12M25/14 (EP) C12M33/00 (EP,CN) C12N5/0643 (CN) C12N5/0654 (EP,CN,US) C12N5/0697 (EP,US) A61L2430/02 (EP,US) A61L2430/40 (US) C12N2501/42 (US) C12N2502/1394 (EP) C12N2513/00 (EP,US) C12N2533/18 (EP) C12N2533/30 (EP,US) C12N2533/54 (US) C12N2537/10 (EP) A61L27/46, C08L67/04, INV (EP)			

56. 3 3D Culture Vessels Method for Collecting the Spheroids from the Vesel and Co-Culturing Method Using the Vessel

Title	Inventors	Applicants	Publication number	Earliest priority	IPC	CPC	Publication date	Earliest publication	Family number
3 3D Culture Vessels Method for Collecting the Spheroids from the Vesel and Co-Culturing Method Using the Vessel	KIM SUNG HWAN	CELLSMITH INC [KR]	KR20220016661A	2020-08-03	C12M1/12 C12M3/00 C12N5/00	C12M21/08 (KR) C12M25/01 (KR) C12N5/0062 (KR)	2022-02-10	2022-02-10	080253645

57. Preparation method of tissue organs

Title	Inventors	Applicants	Publication number	Earliest priority	IPC	CPC	Publication date	Earliest publication	Family number
Preparation method of tissue organs	YAO BIN SONG WEI LI ZHAO ZHU DONGZHEN HUANG SHA FU XIAOBING	CHINESE PLA GENERAL HOSPITAL	CN113846050A CN113846050B	2021-08-30	C12N5/071	C12N5/0602 (CN) C12N2513/00 (CN)	2021-12-28 2023-10-27	2021-12-28	078976508

58. NANOFIBER CARDIAC PATCH AND METHODS OF USE THEREOF

Title	Inventors	Applicants	Publication number	Earliest priority	IPC	CPC	Publication date	Earliest publication	Family number
NANOFIBER CARDIAC PATCH AND METHODS OF USE THEREOF	KHAN MAHMOOD [US] POWELL HEATHER [US]	OHIO STATE INNOVATION FOUNDATION [US]	US2023226259A1	2020-06-03	A61L27/48 A61L27/54	A61L27/3834 (EP) A61L27/48 (EP,US) A61L27/54 (EP,US) A61L2300/252 (EP) A61L2300/414 (EP,US) A61L2400/12 (EP,US)	2023-07-20	2021-12-09	078830583

						A61L2430/20 (EP,US) A61L27/48, C08L29/04, INV (EP) A61L27/48, C08L67/04, INV (EP) A61L27/48, C08L75/04, INV (EP) A61L27/48, C08L89/06, INV (EP)			

59. Hydrogel and preparation method and use thereof

Title	Inventors	Applicants	Publication number	Earliest priority	IPC	CPC	Publication date	Earliest publication	Family number
Hydrogel and preparation method and use thereof	ZOU SHIQUAN XING HELIN LI MAN WU PENG PEI ZHENHUA	BEIJING STOMATOLOGICAL HOSPITAL CAPITAL MEDICAL UNIV	CN113713176A CN113713176B	2021-09-02	A61L27/22 A61L27/38 A61L27/50 A61L27/52 A61L27/54 C08F2/48 C08F299/00	A61L27/222 (CN) A61L27/3834 (CN) A61L27/3847 (CN) A61L27/50 (CN) A61L27/52 (CN) A61L27/54 (CN) C08F2/48 (CN) C08F299/00 (CN) A61L2300/412 (CN) A61L2430/02 (CN) A61L2430/40 (CN)	2021-11-30 2022-09-13	2021-11-30	078681013

60. NEW COUMARIN-POLYMER CONJUGATES AND USES THEREOF

Title	Inventors	Applicants	Publication number	Earliest priority	IPC	CPC	Publication date	Earliest publication	Family number
NEW COUMARIN-POLYMER CONJUGATES AND USES THEREOF	ELVASSORE NICOLA [IT] POLI ILARIA [IT] URCIUOLO ANNA [IT]	ONYEL BIOTECH S R L [IT]	US2023165965A1	2020-04-23	A61K47/54 A61K47/60	A61K47/545 (US) A61K47/60 (US) A61L27/18 (EP) A61L27/222 (EP) B33Y70/00 (EP) C08J3/075 (EP) A61L27/18, C08L71/02, INV (EP)	2023-06-01	2021-10-28	072178889

61. 3D bionic biological scaffold containing stem cell exosomes and application

Title	Inventors	Applicants	Publication number	Earliest priority	IPC	CPC	Publication date	Earliest publication	Family number
3D bionic biological scaffold containing stem cell exosomes and application	AO YINGFANG LI QI HU XIAOQING	UNIV PEKING 3RD HOSPITAL PEKING UNIV 3RD CLINICAL MEDICAL COLLEGE	CN113398332A CN113398332B	2021-08-20	A61L27/20 A61L27/22 A61L27/36 A61L27/54 B33Y70/10	A61L27/20 (CN) A61L27/222 (CN) A61L27/3604 (CN) A61L27/3608 (CN) A61L27/3612 (CN) A61L27/3654 (CN) A61L27/54 (CN) B33Y70/10 (CN) A61L2300/30	2021-09-17 2022-01-11	2021-09-17	077689058

						(CN) A61L2300/412 (CN) A61L2300/602 (CN) A61L2430/06 (CN) A61L2430/24 (CN) A61L27/20, C08L5/08, INV (CN) A61L27/222, C08L89/00, INV (CN)			

62. Bio-ink for treating bone injury and preparation method thereof

Title	Inventors	Applicants	Publication number	Earliest priority	IPC	CPC	Publication date	Earliest publication	Family number
Bio-ink for treating bone injury and preparation method thereof	AO YINGFANG LI QI HU XIAOQING	UNIV PEKING 3RD HOSPITAL PEKING UNIV 3RD CLINICAL MEDICAL COLLEGE	CN113403267A CN113403267B	2021-08-20	C12N5/077 C12N5/0775	C12N5/0654 (CN) C12N5/0655 (CN) C12N5/0662 (CN) C12N5/0667 (CN) C12N2533/54 (CN) C12N2533/80 (CN) C12N2533/90 (CN)	2021-09-17 2022-01-11	2021-09-17	077689068

63. Bio-ink containing stem cell exosome and preparation method thereof

Title	Inventors	Applicants	Publication number	Earliest priority	IPC	CPC	Publication date	Earliest publication	Family number
Bio-ink containing stem cell exosome and preparation method thereof	AO YINGFANG LI QI HU XIAOQING	UNIV PEKING 3RD HOSPITAL PEKING UNIV 3RD CLINICAL MEDICAL COLLEGE	CN113403268A CN113403268B	2021-08-20	C12N5/077 C12N5/0775	C12N5/0655 (CN) C12N5/0662 (CN) C12N5/0667 (CN) C12N2533/54 (CN) C12N2533/80 (CN) C12N2533/90 (CN)	2021-09-17 2022-01-07	2021-09-17	077689020

64. COMPOSITIONS AND METHODS FOR IN SITU-FORMING GELS FOR WOUND HEALING AND TISSUE REGENERATION

Title	Inventors	Applicants	Publication number	Earliest priority	IPC	CPC	Publication date	Earliest publication	Family number
COMPOSITIONS AND METHODS FOR IN SITU-FORMING GELS FOR WOUND HEALING AND TISSUE REGENERATION	MYUNG DAVID [US] HAHN SEI KWANG [US] MADL AMY C [US]	UNIV LELAND STANFORD JUNIOR [US] US GOV VETERANS AFFAIRS [US]	US2023040418A1	2020-02-24	A61F2/14 A61K31/25 A61K31/728 A61K35/30 C07K5/107	A61F2/145 (US) A61K31/25 (US) A61K31/728 (US) A61K35/30 (US) A61K47/36 (EP) A61K9/0048 (EP) A61K9/0051 (EP) C07K5/1016 (US)	2023-02-09	2021-09-02	077491544

65. Novel composite hydrogel stent prepared by 3D biological printing technology and application of novel composite hydrogel stent

Title	Inventors	Applicants	Publication number	Earliest priority	IPC	CPC	Publication date	Earliest publication	Family number
Novel composite hydrogel stent prepared by 3D biological printing technology and application of novel composite hydrogel	LI YAN XIE WEIKE CUI ZHENHUA ZANG HONGYUN LIN ZHAOYI	UNIV SUN YAT SEN	CN113181419A CN113181419B	2021-03-23	A61L26/00 B33Y70/10 C08B37/04	A61L26/0004 (CN) A61L26/0023 (CN) A61L26/0038 (CN)	2021-07-30 2022-08-02	2021-07-30	076973708

stent						A61L26/0047 (CN) A61L26/0061 (CN) A61L26/008 (CN) A61L26/009 (CN) B33Y70/10 (CN) C08B37/0084 (CN) A61L2300/412 (CN) A61L26/0023, C08L5/04, INV (CN) A61L26/0047, C08L89/00, INV (CN)			

66. Biomaterials for the prevention and the treatment of tissue disorders

Title	Inventors	Applicants	Publication number	Earliest priority	IPC	CPC	Publication date	Earliest publication	Family number
Biomaterials for the prevention and the treatment of tissue disorders	DUFRANE DENIS [BE]<br>THEYS NICOLAS [BE]	NOVADIP BIOSCIENCES [BE]	US2024216572A1	2019-11-29	A61L27/10<br>A61L27/12<br>A61L27/26<br>A61L27/36<br>A61L27/38<br>C08L29/04<br>C08L31/06<br>C08L39/06<br>C08L5/04<br>C08L5/08<br>C08L5/12<br>C08L67/04<br>C08L89/06	A61K35/00 (EP)<br>A61K35/12 (EP,IL,KR)<br>A61K35/28 (EP,IL,KR)<br>A61K35/32 (EP,IL,KR)<br>A61L27/10 (US)<br>A61L27/12 (US)<br>A61L27/222 (EP,IL,KR)<br>A61L27/26 (US)<br>A61L27/3633 (EP,IL,KR,US)<br>A61L27/38 (US)<br>A61L27/3834 (EP,IL,KR)<br>A61L27/46 (EP,IL,KR)<br>A61L27/48 (EP,IL,KR)<br>C08L29/04 (US)<br>C08L31/06 (US)<br>C08L39/06 (US)<br>C08L5/04 (US)<br>C08L5/08 (US)<br>C08L5/12 (US)<br>C08L67/04 (US)<br>C08L89/06 (IL,US)<br>C12N5/0075 (KR)<br>C12N5/0654 (EP,IL,KR)<br>C12N5/0667 (EP,IL,KR)<br>A61L2300/414 (EP,IL,KR,US)	2024-07-04	2021-06-03	073598138

						A61L2400/12 (EP,IL,KR) A61L2430/34 (US) C12N2500/38 (EP,IL) C12N2501/999 (EP,IL,KR) C12N2506/1384 (EP,IL,KR) C12N2513/00 (EP,IL,KR) C12N2523/00 (EP,IL,KR) C12N2529/10 (EP,IL,KR) C12N2533/18 (EP,IL,KR) C12N2533/54 (EP,IL,KR) C12N2533/90 (EP,IL,KR) A61L27/48, C08L89/06, INV (EP)			

67. コアシェル繊維プリントのシステム及び方法

Title	Inventors	Applicants	Publication number	Earliest priority	IPC	CPC	Publication date	Earliest publication	Family number
コアシェル繊維プリントのシステム及び方法			JP2023500102A	2019-11-01	B29C64/106 B29C64/209 B33Y10/00 B33Y30/00	B29C64/106 (EP) B29C64/118 (EP,KR,US) B29C64/209 (EP,KR,US) B29C64/393 (US) B33Y10/00 (EP,KR) B33Y30/00 (EP,KR) B33Y70/00 (EP,KR) B33Y10/00 (US) B33Y30/00 (US) B33Y50/02 (US) B33Y70/00 (US)	2023-01-04	2021-05-06	075714886

68. Kit and method for development of spheroids

Title	Inventors	Applicants	Publication number	Earliest priority	IPC	CPC	Publication date	Earliest publication	Family number
Kit and method for development of spheroids	GATENHOLM ERIK [SE] MARTINEZ HECTOR [SE] NAMRO REDWAN ITEDAL [SE] BLELL JOSEFIN [SE]	CELLINK AB [SE]	SE1950925A1	2019-08-13	A61L27/24 A61L27/36 A61L27/38 C12N5/071 A61L27/14 A61L27/54 B21C23/00 B33Y10/00 B33Y70/00 B33Y80/00	A61L27/24 (SE) A61L27/3633 (SE) A61L27/3886 (SE) B33Y70/10 (SE) C12N5/0062 (SE) A61L27/14 (SE) A61L27/26 (SE) A61L27/3683 (SE) A61L27/54	2021-02-14	2021-02-14	074849289

						(SE) B21C23/00 (SE) B33Y10/00 (SE) B33Y80/00 (SE) C12N2501/999 (SE) C12N2503/04 (SE) C12N2533/50 (SE) C12N2533/54 (SE) C12N2533/90 (SE)			

69. HYALURONIC ACID AND GELATIN-CONTAINING FORMULATIONS

Title	Inventors	Applicants	Publication number	Earliest priority	IPC	CPC	Publication date	Earliest publication	Family number
HYALURONIC ACID AND GELATIN-CONTAINING FORMULATIONS	BHOWMICK TUHIN [US] CHANDRU ARUN [IN] SELVAM SHIVARAM [IN] AGRAWAL PARINITA [IN] BEN THOMAS MIDHUN [IN] BELLUR PRAYAG [IN] MENON DEEPTHI [IN]	PANDORUM TECH PRIVATE LIMITED [IN]	US2022218873A1	2019-07-26	A61L27/20 A61L27/22 A61L27/38 B33Y10/00 B33Y70/10 B33Y80/00	A61K35/28 (KR) A61K35/30 (US) A61L27/20 (US) A61L27/222 (US) A61L27/24 (US) A61L27/26 (EP,GB,KR,US) A61L27/3834 (EP,GB,KR,US) A61P27/02 (US) B29C64/106 (US) B33Y10/00 (EP,GB,KR,US) B33Y70/00 (EP,GB,KR,US) B33Y70/10 (US) B33Y80/00 (EP,GB,KR,US) C08L5/08 (KR) C08L89/06 (KR) C09D11/03 (KR) C09D11/04 (KR) C12N5/0621 (KR) G01N33/5058 (US) G01N33/5088 (US) A61K35/28 (EP,GB,US) A61L2430/16 (EP,GB,KR,US) B29K2005/00 (US) B29K2077/00 (US) B29L2031/7532 (US) C12N2502/1352 (EP,GB,KR) C12N2513/00 (EP,GB,KR)	2022-07-14	2021-02-04	074228673

						C12N2533/54 (EP,GB,KR) C12N2533/80 (EP,GB,KR) C12N5/0621 (EP,GB) A61L27/26, C08L5/08, INV (EP) A61L27/26, C08L89/06, INV (EP)			

70. DECELLULARIZED MATRIX HYDROGEL, PREPARATION METHOD THEREFOR, AND APPLICATION THEREOF

Title	Inventors	Applicants	Publication number	Earliest priority	IPC	CPC	Publication date	Earliest publication	Family number
DECELLULARIZED MATRIX HYDROGEL, PREPARATION METHOD THEREFOR, AND APPLICATION THEREOF	GAO YI [CN] YI XIAO [CN] LI YANG [CN]	GUANGDONG UNISUN BIOTECHNOLOGY CO LTD [CN]	WO2022042704A1	2020-08-28	A61K47/46 A61K9/06 A61L27/36 A61L27/52	A61K47/46 (CN) A61K9/06 (CN) A61L27/3604 (CN) A61L27/3687 (CN) A61L27/52 (CN)	2022-03-03	2020-12-18	073758214

71. 3D Bioprinted Skin Tissue Model

Title	Inventors	Applicants	Publication number	Earliest priority	IPC	CPC	Publication date	Earliest publication	Family number
3D Bioprinted Skin Tissue Model	BONDESSON ISABELLA [SE] NAMRO REDWAN ADEL ITEDAL [SE] MARTINEZ HECTOR [SE] GATENHOLM ERIK [SE]	CELLINK AB [SE]	US2022249738A1	2019-06-13	A61L27/20 A61L27/38 A61L27/44 A61L27/60 C12N5/00 C12N5/071	A61F2/105 (SE) A61L27/20 (EP,SE,US) A61L27/22 (EP) A61L27/3804 (EP,US) A61L27/44 (EP,US) A61L27/60 (EP,SE,US) B33Y70/00 (EP) B33Y70/10 (SE) B33Y80/00 (EP) C12M21/08 (SE) C12N5/0062 (EP,SE,US) C12N5/0697 (EP) C12N5/0698 (SE,US) G09B23/306 (EP) B29C64/10 (SE) B33Y10/00 (SE) B33Y30/00 (SE) B33Y80/00 (SE) C12N2503/02 (EP,US) C12N2503/04 (EP) C12N2503/06 (US) C12N2506/45 (EP) C12N2513/00	2022-08-11	2020-12-14	071094372

						(EP,SE,US) C12N2533/52 (EP,US) C12N2533/54 (EP,US) C12N2533/74 (EP,US)			

72. TREATMENT OF KIDNEY DISEASE IN SUBJECTS WITH KIDNEY AND/OR URINARY TRACT ANOMALIES

Title	Inventors	Applicants	Publication number	Earliest priority	IPC	CPC	Publication date	Earliest publication	Family number
TREATMENT OF KIDNEY DISEASE IN SUBJECTS WITH KIDNEY AND/OR URINARY TRACT ANOMALIES	BERTRAM TIMOTHY [KY] JAIN DEEPAK [US]	BERTRAM TIMOTHY [KY] JAIN DEEPAK [US]	US2022202867A1	2019-05-02	A61K35/22 A61P13/12	A61K35/22 (EP,IL,KR,US) A61P13/12 (EP,IL,KR,US)	2022-06-30	2020-11-05	073029546

73. Liver Tissue Model Constructs and Methods for Providing the Same

Title	Inventors	Applicants	Publication number	Earliest priority	IPC	CPC	Publication date	Earliest publication	Family number
Liver Tissue Model Constructs and Methods for Providing the Same	NGUYEN DUONG [SE] NAMRO REDWAN ADEL ITEDAL [SE] MARTINEZ HECTOR [SE] GATENHOLM ERIK [SE]	CELLINK AB [SE]	US2022145259A1	2019-03-13	A61L27/26 A61L27/36 A61L27/38 A61L27/48 B33Y10/00 B33Y40/10 B33Y70/00 B33Y80/00 C12N5/00 C12N5/071	A61L27/26 (US) A61L27/3633 (US) A61L27/3641 (US) A61L27/3804 (US) A61L27/3834 (US) A61L27/3839 (US) A61L27/3886 (US) A61L27/48 (US) B33Y10/00 (US) B33Y40/10 (US) B33Y70/00 (US) B33Y80/00 (US) C12N5/0062 (EP,US) C12N5/0671 (EP,US) C12N5/0697 (EP) A61L2300/414 (US) A61L2300/802 (US) A61L2430/28 (US) C12N2503/02 (EP) C12N2503/04 (EP,US) C12N2506/45 (EP) C12N2513/00 (EP) C12N2533/52 (EP,US) C12N2533/54	2022-05-12	2020-09-17	069846084

						(EP,US) C12N2533/74 (EP,US) C12N2533/78 (US)			

74. SELF-ASSEMBLING GRAPHENE OXIDE-PROTEIN MATRIX

Title	Inventors	Applicants	Publication number	Earliest priority	IPC	CPC	Publication date	Earliest publication	Family number
SELF-ASSEMBLING GRAPHENE OXIDE-PROTEIN MATRIX	WU YUANHAO [GB] WANG WEN [GB] CHAVARRIA ALVARO MATA [GB]	UNIV LONDON QUEEN MARY [GB]	US2021346570A1	2018-09-19	A61L27/08 A61L27/22 A61L27/38 A61L27/50 B33Y10/00 B33Y80/00	A61L27/08 (EP,US) A61L27/227 (EP,US) A61L27/3808 (EP,US) A61L27/507 (EP,US) B33Y10/00 (EP,US) B33Y70/00 (EP) B33Y80/00 (EP,US) A61L2300/214 (US) A61L2300/414 (US) A61L2300/622 (US) A61L2300/624 (US) A61L2430/40 (US)	2021-11-11	2020-03-26	064013402

75. COMPOSITIONS COMPRISING CELL-DELIVERED VESICLES AND USES THEREOF

Title	Inventors	Applicants	Publication number	Earliest priority	IPC	CPC	Publication date	Earliest publication	Family number
COMPOSITIONS COMPRISING CELL-DELIVERED VESICLES AND USES THEREOF	BERTRAM TIMOTHY A [US] JAIN DEEPAK [US]	BERTRAM TIMOTHY A [US] JAIN DEEPAK [US]	US2021386786A1	2018-08-31	A61K35/22 C12N5/071 G01N33/50 G01N33/68	A61K31/7105 (KR) A61K31/713 (EP) A61K35/22 (EP,KR,US) A61K9/0019 (EP,KR) A61K9/19 (EP) A61P13/12 (KR) A61P25/00 (EP) C12N5/0687 (EP,KR,US) C12Q1/6883 (KR) G01N33/5076 (EP,KR,US) G01N33/68 (EP) G01N33/6803 (US) G01N33/6872 (KR) A61K38/00 (EP) C12N2310/141 (EP) C12N2500/25 (EP,KR)	2021-12-16	2020-03-05	069644587

						C12N2500/99 (EP,KR) C12N2501/11 (EP,KR,US) C12N2501/12 (EP,KR,US) C12N2502/256 (EP,KR,US) C12Q2600/118 (KR) C12Q2600/178 (KR) G01N2333/70596 (EP) G01N2800/347 (EP,KR) G01N2800/52 (KR)			

76. NOVEL METHOD TO ENGINEER TRANSLANTABLE HUMAN TISSUES

Title	Inventors	Applicants	Publication number	Earliest priority	IPC	CPC	Publication date	Earliest publication	Family number
NOVEL METHOD TO ENGINEER TRANSLANTABLE HUMAN TISSUES	ZHANG KUN [US] WU YAN [US] DAILAMY AMIR [US] MALI PRASHANT [US] MCDONALD DANIELLA [US] PAREKH UDIT [US] HU MICHAEL [US]	UNIV CALIFORNIA [US]	US2022235104A1	2018-07-06	A61K31/522 A61K45/06 C07K14/47 C12N15/11 C12N15/86 C12N5/071 C12N9/22	A61K31/522 (US) A61K35/545 (EP) A61K45/06 (US) B33Y10/00 (EP) B33Y80/00 (EP) C07K14/4702 (US) C12N15/111 (US) C12N15/63 (EP) C12N15/86 (US) C12N5/0062 (EP) C12N5/0606 (EP) C12N5/0693 (EP) C12N5/0697 (EP,US) C12N9/22 (US) B29C64/106 (EP) B29C64/118 (EP) B29L2031/7532 (EP) C12N2310/20 (EP,US) C12N2501/65 (EP) C12N2506/02 (EP) C12N2506/1353 (EP) C12N2506/28 (EP) C12N2506/45 (EP) C12N2510/00 (EP) C12N2310/141, C12N2330/10, ADD (EP)	2022-07-28	2020-01-09	069059826

77. CELL CULTURING DEVICE AND METHOD

Title	Inventors	Applicants	Publication number	Earliest priority	IPC	CPC	Publication date	Earliest publication	Family number
CELL CULTURING DEVICE AND METHOD	DEUTSCH MORDECHAI [IL] ZURGIL NAOMI [IL] AFRIMZON ELENA [IL] MOSHKOV SERGEI [IL] RAVID-HERMESH ORIT [IL] SHAFRAN YANA [IL] SOBOLEV MARIA [IL]	UNIV BAR ILAN [IL]	US2020399571A1	2018-01-23	C12M1/00 C12M1/12 C12M1/32 C12N5/00	C12M23/12 (EP,KR,US) C12M23/20 (EP,KR) C12M23/22 (US) C12M25/04 (EP) C12M25/06 (EP,KR) C12M25/14 (EP,US) C12N5/0062 (US) C12N5/0075 (US) C12N2513/00 (US) C12N2533/90 (KR)	2020-12-24	2019-08-01	067395268

78. POROUS IMPLANTABLE DEVICES

Title	Inventors	Applicants	Publication number	Earliest priority	IPC	CPC	Publication date	Earliest publication	Family number
POROUS IMPLANTABLE DEVICES	SON ALEXANDER I [US] HASHIMOTO-TORII KAZUE [US] TORII MASAAKI [US] MORTON PAUL D [US] ISHII SEIJI [US] OPFERMANN JUSTIN [US] LIU JUDY [US] FISHER JOHN [US] SANTORO MARCO [US] KIM PETER C W [US]	CHILDRENS NAT MEDICAL CT [US] UNIV MARYLAND [US]	US11534283B2 US2020289250A1	2017-09-06	A61F2/00 A61F2/02 A61K35/30 A61K38/17 A61L31/04 A61L31/14	A61F2/0077 (EP,US) A61F2/022 (US) A61K35/30 (EP,US) A61K38/1767 (US) A61L31/005 (EP) A61L31/048 (US) A61L31/146 (EP,US) A61L31/16 (EP) A61F2002/0081 (EP,US) A61F2210/0095 (US) A61F2230/0069 (US) A61F2250/0067 (EP,US) A61L2300/252 (EP) A61L2300/258 (EP)	2020-09-17 2022-12-27	2019-03-14	065635206

79. IMMUNOPRIVILEGED BIOACTIVE RENAL CELLS FOR THE TREATMENT OF KIDNEY DISEASE

Title	Inventors	Applicants	Publication number	Earliest priority	IPC	CPC	Publication date	Earliest publication	Family number
IMMUNOPRIVILEGED BIOACTIVE RENAL CELLS FOR THE TREATMENT OF KIDNEY DISEASE	BERTRAM TIMOTHY A [KY] JAIN DEEPAK [US] BASU JOYDEEP [US] LUDLOW JOHN W [US]	BERTRAM TIMOTHY A [KY] JAIN DEEPAK [US]	US2020216816A1	2017-06-21	A61K35/22 A61K47/69 A61K9/00 C12N15/85 C12N5/071	A61K35/22 (EP,KR,US) A61K47/42 (EP,KR) A61K47/6903 (US) A61K9/0019 (EP,KR,US) A61K9/06 (EP,KR) A61P13/12 (EP,KR)	2020-07-09	2018-12-27	064737306

						C07K14/70539 (EP,KR) C12N15/85 (US) C12N5/0686 (EP,KR,US) C12N9/22 (EP,KR) C12N2500/25 (EP) C12N2501/11 (EP) C12N2501/12 (EP) C12N2510/00 (EP,US) C12N2533/54 (EP) C12N2539/10 (EP)			

80. INJECTABLE CELL AND SCAFFOLD COMPOSITIONS

Title	Inventors	Applicants	Publication number	Earliest priority	IPC	CPC	Publication date	Earliest publication	Family number
INJECTABLE CELL AND SCAFFOLD COMPOSITIONS	JAIN DEEPAK [US] BERTRAM TIMOTHY A [US]	JAIN DEEPAK [US] BERTRAM TIMOTHY A [US]	US2020405913A1	2017-03-31	A61L27/24 A61L27/36 A61L27/38 A61L27/52 A61L27/54	A61K35/12 (EP,KR) A61K35/22 (KR) A61K9/0019 (KR) A61L27/222 (EP,KR) A61L27/24 (US) A61L27/3604 (US) A61L27/3633 (EP,KR) A61L27/3804 (EP,KR) A61L27/3813 (US) A61L27/52 (US) A61L27/54 (US) A61L27/56 (KR) A61P13/12 (EP,KR) A61L2400/06 (EP,KR) A61L2430/26 (KR) A61L27/00 (EP) A61L31/00 (EP)	2020-12-31	2018-10-04	063676750

81. METHODS FOR IDENTIFYING AND ISOLATING CARDIAC STEM CELLS AND METHODS FOR MAKING AND USING THEM

Title	Inventors	Applicants	Publication number	Earliest priority	IPC	CPC	Publication date	Earliest publication	Family number
METHODS FOR IDENTIFYING AND ISOLATING CARDIAC STEM CELLS AND METHODS FOR MAKING AND USING THEM	SUSSMAN MARK ALAN [US]	SAN DIEGO STATE UNIV SDSU FOUNDATION DBA SAN DIEGO STATE UNIV RESEARCH FOUNDATION [US]	US2020190475A1	2017-02-23	A61K35/34 C12N5/077 C12Q1/6841 C12Q1/6851 C12Q1/686	A61K35/34 (EP,US) C12N5/0657 (EP,US) C12Q1/6841 (US) C12Q1/6851 (US) C12Q1/686 (US)	2020-06-18	2018-08-30	063253400

						C12Q2561/113 (US) C12Q2600/158 (US)			

82. COMPOSITIONS AND METHODS OF TREATING CANCER

Title	Inventors	Applicants	Publication number	Earliest priority	IPC	CPC	Publication date	Earliest publication	Family number
COMPOSITIONS AND METHODS OF TREATING CANCER	AVIGAN DAVID [US] ROSENBLATT JACALYN [US] KUFE DONALD [US]	DANA FARBER CANCER INST INC [US] BETH ISRAEL DEACONESS MEDICAL CT INC [US]	US2019269775A1	2016-11-14	A61K31/4035 A61K31/454 A61K31/7068 A61K31/708 A61K31/7125 A61K35/13 A61K35/15 A61K39/08 A61K39/395 A61K45/06 C12N5/16	A61K31/4035 (US) A61K31/454 (US) A61K31/7068 (US) A61K31/708 (US) A61K31/7125 (US) A61K35/13 (US) A61K35/15 (EP,US) A61K39/0011 (EP,US) A61K39/08 (US) A61K39/39541 (US) A61K45/06 (US) C12N5/16 (EP,US) A61K2035/122 (US) A61K2039/5152 (EP,US) A61K2039/5154 (EP,US) C12N2501/052 (EP,US) C12N2501/056 (EP,US)	2019-09-05	2018-05-17	060480466

83. IN VIVO LIVE 3D PRINTING OF REGENERATIVE BONE HEALING SCAFFOLDS FOR RAPID FRACTURE HEALING

Title	Inventors	Applicants	Publication number	Earliest priority	IPC	CPC	Publication date	Earliest publication	Family number
IN VIVO LIVE 3D PRINTING OF REGENERATIVE BONE HEALING SCAFFOLDS FOR RAPID FRACTURE HEALING	VARANASI VENU G [US] ILYAS AZHAR [US] KRAMER PHILIP ROGER [US] AZIMAIE TAHA [US] ASWATH PRANESH B [US] CEBE TUGBA [US]	TEXAS A & M UNIV SYS [US] UNIV TEXAS [US]	US10442182B2 US2017143831A1	2015-11-24	A61K9/14 B29C67/00 B33Y30/00 A61K31/722 A61K33/00 A61K38/39 A61K41/00 A61N5/06 B33Y10/00 B33Y70/00 B29C64/106 B33Y80/00 B29K67/00	A61K31/722 (EP,US) A61K33/00 (EP,US) A61K38/39 (EP,US) A61K41/00 (EP,US) A61N5/062 (US) B29C64/106 (EP) B29C64/118 (EP,US) B29C64/307 (EP,US) B33Y10/00 (EP,US) B33Y70/10 (EP,US) B33Y80/00 (EP,US) A61N2005/0661	2017-05-25 2019-10-15	2017-05-25	058719942

						(US) B29K2067/046 (US) B29K2995/0056 (US)			

84. HYDROGEL COMPOSITIONS COMPRISING ENCAPSULATED CELLS AND METHODS OF USE THEREOF

Title	Inventors	Applicants	Publication number	Earliest priority	IPC	CPC	Publication date	Earliest publication	Family number
HYDROGEL COMPOSITIONS COMPRISING ENCAPSULATED CELLS AND METHODS OF USE THEREOF	SHIN JAE-WON [US] MAO ANGELO S [US] UTECH STEFANIE [US] WEITZ DAVID A [US] MOONEY DAVID J [US] UZUN OKTAY [US]	HARVARD COLLEGE [US]	US11229607B2 US2017196818A1	2014-06-30	A61K35/28 A61K9/00 A61K9/48 A61K35/12 A61K9/50 C12N11/04 C12N11/10	A61K35/12 (EP,US) A61K35/28 (EP,US) A61K9/0019 (EP,US) A61K9/4816 (US) A61K9/5036 (EP,US) A61K9/5052 (EP,US) C12N11/04 (EP,US) C12N11/10 (EP,US) A61K2035/124 (US)	2017-07-13 2022-01-25	2016-01-07	055019926
